# Interventions to identify and manage depression delivered by ‘nontraditional’ providers to community‐dwelling older adults: A realist review

**DOI:** 10.1111/hex.13594

**Published:** 2022-09-06

**Authors:** Tom Kingstone, Carolyn A. Chew‐Graham, Nadia Corp

**Affiliations:** ^1^ School of Medicine Keele University Staffordshire UK; ^2^ Research and Innovation Department Midlands Partnership NHS Foundation Trust Stafford UK

**Keywords:** extended roles, mental health, older people, programme theory, public services, realist synthesis

## Abstract

**Background:**

Mental health problems experienced by older adults (60+ years of age) may remain hidden due to individual and system‐level barriers. Opportunities to support early identification and management are therefore crucial. The National Health Service recommends wider public services that are embedded within local communities, but are not traditionally part of the healthcare landscape (i.e., ‘nontraditional’), could facilitate engagement with healthcare by members of the public. Evidence for interventions involving Fire and Rescue, Police, Library services and postal workers, as nontraditional providers of mental health services, has not been synthesized previously. This review aims to understand how, why and in what contexts mental health interventions delivered by these nontraditional providers, to older adults, work.

**Methods:**

A realist review of interventions to identify and/or manage mental health problems (depression with or without anxiety) experienced by older adults. Systematic, cluster and iterative literature searches were conducted. Intervention evidence was appraised for rigour and explanatory relevance and then coded to inform context‐mechanism‐outcome configurations (CMOCs). A public advisory group supported our initial evidence search strategy and definition of key terms. This review is registered with PROSPERO (CRD42020212498).

**Results:**

Systematic searches revealed a dearth of evidence reporting mental health interventions delivered by nontraditional providers. Our scope was adjusted to consider interventions delivered by Fire and Police services only and for wider health and wellbeing concerns (e.g., dementia, falls prevention, mental health crises). Forty‐three pieces of evidence were synthesized. Key themes included: legitimizing expanded roles, focusing on risk, intervention flexibility and organization integration; further subthemes are described. Themes map onto CMOCs and inform a preliminary programme theory. Findings were transposed to mental health contexts.

**Conclusions:**

Findings highlight challenges and opportunities for Fire and Police services, as nontraditional providers, to deliver interventions that identify and/or manage mental health problems among older adults. Our programme theory explains what *could* work, how, for whom and also by whom (i.e., which public services). Further empirical evidence is needed to test interventions, understand acceptability and inform implementation.

**Patient or Public Contribution:**

A public advisory group comprising older adults with lived experience of mental health problems and informal caregivers contributed to the original application, reviewed the scope and informed the approach to dissemination.

## 1 BACKGROUND

Mental ill‐health is a leading cause of disability worldwide.^1^, ^2^ One in four older adults experience symptoms of mental ill‐health with multiple forms of loss identified as key contributing factors, including a decline in or loss of social connections, role, identity, physical health and cognitive capacity.^3^, ^4^, ^5^, ^6^ The COVID‐19 pandemic has exacerbated many of these factors for older adults with multimorbidity.^7^ However, fewer than one in six older adults consult a healthcare professional about their symptoms.^3^ Barriers to help‐seeking are reported, including stigma, lack of mental health awareness and unwillingness to disclose symptoms, which may represent mental ill‐health.^8^ Older adults continue to be underrepresented in terms of engagement with Improving Access to Psychological Therapies (IAPTs), as evidenced by lower rates of referral and access to treatment.^9^ In 2014/2015, only 7% of people completing treatment provided by IAPT were 65 years and above.^10^, ^11^ Inequalities linked to deprivation and rurality may also restrict access to mental health services by older adults.^12^ Without adequate management, mental ill‐health can negatively impact morbidity and mortality.^13^

To improve access to care, National Health Service (NHS) England encourages wider public services to ‘break out’ from their perceived boundaries.^14^, ^15^ This shift is reflected in local government policy discourse, as evidenced by the ‘Health in All Policies’ approach.^16^ Fire and Rescue, Police and Library services have been identified as key health assets on the basis of their existing points of contact with members of the public out in the community, as evidenced by key consensus statements.^17^, ^18^ In relation to mental health, NHS policy focuses on early detection of problems—‘Five Year Forward View for Mental Health’^19^—and supports the emergence of such ‘nontraditional’ providers of community‐based healthcare (i.e., public services and individuals not traditionally viewed as part of the healthcare landscape)—‘NHS Long Term Plan’.^20^

Evidence suggests that task‐shifting in the context of mental health diagnosis, assessment and treatment by nonspecialists can be effective in strengthening mental healthcare systems.^21^ Evidence for nonspecialist roles in mental health has been synthesized in a variety of different contexts, including: community pharmacy regarding the management of depression in adults,^22^ mental health medication support^23^ and depression screening^24^; lay health workers delivering psychological interventions in high income,^25^ low‐ and middle‐income countries^21^; and social prescribing in primary care.^26^, ^27^ However, evidence for interventions that target older adults is limited and potential providers, such as Fire and Rescue, Police, Library and Postal services, have been largely overlooked despite the opportunity for contact with older adults these services may offer as health assets.

Fire and Rescue services operating in some English regions have delivered public health interventions (e.g., smoking cessation, flu vaccinations, falls prevention) and it is widely recognized that mental health problems are a risk factor for fire‐related accidents and fatalities.^28^ Fire and Rescue Services with community fire prevention roles are likely to come into contact with vulnerable members of the public at higher risk of mental health problems, such as older adults with long‐term health conditions^8^—as such, mental health could be an important factor in supporting the targeting of fire prevention operations. Police forces in England have trialled mental health triage interventions, as officers are often first‐on‐the‐scene to public incidents involving mental health problems; however, a rapid synthesis indicates methodological weaknesses.^29^ Postal workers have also led initiatives to prevent and/or reduce loneliness; trials in the United Kingdom are reported as part of the Royal Mail's ‘feet on the street’ campaign.^30^ The evidence base for these types of nontraditional providers to identify and/or manage mental health problems is currently unclear.

This paper reports a realist review to synthesize this evidence and establish a theoretical framework to ascertain how, for whom and in what circumstances, interventions to identify and/or manage depression delivered by nontraditional providers in the public sector, to older adults, work. To understand the phenomenon, the review aimed to:

1. Describe current empirical evidence for interventions that identify and/or manage depression (with or without anxiety) delivered by nontraditional providers (including Fire and Rescue Service, Police, Library services and postal workers) to older people (60+ years) in noninstitutional settings.
2. Develop an understanding of the mechanisms at work (i.e., interactions, relationships, identities, values) between these nontraditional providers, receivers and traditional health service providers

## 2 METHODS

### 2.1 Rationale

Realist reviews are theory‐driven and interpretive with the purpose of identifying and articulating the underpinning mechanisms that explain how, why and when an intervention(s) works.^31^, ^32^ Realist theories commonly utilize the heuristic device of context‐mechanism‐outcome configurations (CMOCs), or similar, to describe how certain social contexts and causal mechanisms (i.e., how an individual may respond to an offer of a resource or aspect of an intervention) generate particular outcomes.^33^, ^34^ A realist review was considered the most appropriate approach for this evidence synthesis, as the interventions were anticipated to be complex and informed by evidence that a traditional systematic review would exclude on methodological grounds.

This review was guided by the five key stages outlined by Pawson^34^: (i) clarification of scope, (ii) searching for evidence, (iii) selection, appraisal and data extraction of documents, (iv) evidence synthesis and conclusions and (v) dissemination, implementation and evaluation. Four key assumptions informed the approach to this review: accepting that information is fragmented; recognizing that large amounts of data are required to theory test; acknowledging that theory testing is unpredictable, unstable and uncertain; and accepting that data collection is iterative.^35^ Evidence from a wide range of documentary sources (including peer‐review journals, grey literature, policy documents) and expert opinion were considered.^36^

The Realist And Meta‐narrative Evidence Syntheses: Evolving Standards publication standards were followed.^37^ Table 1 provides definitions of key terms to support reading of our approach and comparison.

**Table 1:** Glossary of realist review terms

Context	Aspects of the physical and social environment that are relevant to explanatory statements about why, how and for whom a programme works (e.g., demographics, legislation, cultural norms, historical events, current events, economic conditions, geography). Contextual factors could involve elements at any level micro‐, meso‐ and/or macrolevel.
Mechanism	Taken to include two connected elements, resources and responses, in the delivery and receipt of an intervention:
	(1) Resources—These may include: information, advice, trust, engagement and motivation but not necessarily material resources but also opportunities to do X, Y or Z.
	(2) Responses—The cognitive and/or emotional way that people reason or respond to resources (e.g., the offer of Resource A *enables* receivers to…)
Outcomes	Intended or unintended impacts of the context‐mechanism relationship, that is, what was the result? Types of outcomes include: health outcomes, services‐oriented outcomes, uptake outcomes and sustainability outcomes. Outcomes are often measurable (e.g., primary and secondary trial outcomes) but qualitative outcomes that offer ontological depth are included.
Program theory	This is a hypothesis about a specific programme and how it may work, for example, how resources are expected to trigger specific responses and outcomes in specific contexts. Theoretical statements help to describe the logic of a programme and will use the ‘If… Then… Because…’ framework.
Middle range theory	Theory at this level provides a broad level explanation of social phenomena that moves beyond individual accounts of behaviour and experience (i.e., of those ‘on the ground’). A middle‐range theory maintains a connection with empirical data and considers one or a small number of concepts (e.g., Normalization Process Theory). As opposed to grand theories, which offer a higher level of abstraction (e.g., Feminist Theory).^30^

### 2.2 Clarification of scope and changes from the original protocol

An a priori protocol was developed and registered with the International prospective register of systematic reviews (PROSPERO, CRD42020212498).

An exploratory background search was conducted in five bibliographic electronic databases, National Institute for Health and Care Excellence Evidence search, GoogleScholar (via Publish or Perish) and two organizational websites (Gov.uk, Her Majesty's Inspectorate of Constabulary and Fire & Rescue Services) on 1 July 2020 (refer to Supporting Information: File S1 for search strategy Appendix S1A,B). Three further organizational websites were also searched (Public Health Scotland, Scotland Police, Scottish Fire and Rescue Service) on 25 August 2020. In addition, 12 previously identified publications were also considered. All study and document types (published and unpublished/grey literature) were considered, irrespective of date, however, only English‐language items were included.

Changes were made from the original protocol, based on the initial background search. The search highlighted a paucity of interventions focused specifically on depression, or more broadly on mental health, in older adults delivered by nontraditional providers. Furthermore, where other health‐related interventions for older adults were identified these predominantly involved either the police and/or fire service. Consequently, the review's scope was (i) broadened to include other health conditions or health‐related risks associated with older adults, which were perceived as potentially stigmatizing, that is, dementia, accidental falls, frailty and vulnerability, and (ii) narrowed to focus specifically on fire and police services. The geographical scope also changed. The initial scope had been to consider evidence from the United Kingdom and other countries that shared similar healthcare contexts, for example, Western Europe, Australia, New Zealand and Canada. However, early searches identified prominent evidence from the United States, this evidence was included based on similarities at a nontraditional service level (e.g., regional fire services or departments with emergency response and community outreach roles).

### 2.3 Patient and public involvement

A patient and public advisory group was convened with older adults (60 years and above), and/or informal caregivers for older adults, with lived experience of a mental health problem. The group attended an online meeting at the start of the review to share views on the design and scope. To enable inclusion and engagement, a role‐play scenario was used to facilitate discussion of an otherwise complex review, and to inform literature searching and the development of initial programme theory statements. The role‐play activity centred upon a hypothetical situation involving an individual in a uniform that knocks at the door; this proceeded to the uniformed person identifying him/herself, asking to enter the home, explaining the main purpose of their visit (linked to the traditional role), then introducing mental health‐related topics (i.e., untraditional role). Advisory group members were asked to share their views on what they would do next and why; this helped to elicit initial ideas on mechanisms at each stage of the hypothetical interaction. Advisory group members identified key barriers and facilitators to engagement, such as prior awareness about the interaction, the limits and boundaries of trust, fear of crime, importance of language about mental health, potential for stigma (e.g., being identified as ‘vulnerable’), potential for domestic violence (e.g., being unable to talk openly) and threat to independence (i.e., inviting unwanted help at home). The group agreed with the scope and inclusion of broader health and wellbeing topics and their relevance in relation to stigma.

### 2.4 Eligibility

Sources of evidence were considered eligible for inclusion if they addressed interventions delivered by the police or fire services alone or in collaboration with partner agencies (e.g., primary care, public health, local authority or voluntary sector services) and targeted the identification and/or management of mental ill health (i.e., depression, low mood, distress, and anxiety), dementia, accidental falls, vulnerability or frailty, in older adults (≥60 years) living independently. If relevant, studies that included any comparator intervention or population were included. Severe mental illness (e.g., schizophrenia, bipolar disorder, psychosis) was beyond the scope of this review and therefore excluded. Also excluded were studies that focused on older adults in residential‐ or nursing‐care homes, or receiving inpatient care; or were conducted in low‐ and middle‐income countries. Of interest were sources that described any aspect of the intervention from its development and implementation by the provider, to its delivery and observed outcomes. English‐language‐only articles were included.

### 2.5 Search methods and study selection

The main search involved a comprehensive search of 14 bibliographic databases (Medline, EMBASE, AMED, HMIC, CINAHLPlus, Academic Search Complete, AgeLine, PsycINFO, PsycARTICLES, Web of Science (Science Citation Index‐Expanded, Social Science Citation Index, Conference Proceedings Citation Index [CPCI]‐Science and CPCI‐Social Science and Humanities), ASSIA, Cochrane Library, SCIE‐Social Care online, and Epistemonikos) from inception until October 2020. Searches were designed by an experienced information specialist (N. C.) and utilized database subject headings and text word searching in the title, abstract or keywords, combining terms for: (1) older adults; (2) depression, dementia, falls, vulnerable or frailty; (3) fire or police service and (4) independent living or intervention (see Supporting Information: File S1). Search terms were adapted as appropriate for each database and platform. Articles were restricted to the English language only, no other limits were set. This was supplemented with a search of grey literature (OpenGrey, NDLTD, EThOS, Nuffield Trust, Mind, Age UK, Mental Health Foundation and Alzheimer's Society UK).

Results from all searches were imported into EndNote X9 (reference management software, Clarivate Analytics. Available at https://endnote.com/) and duplicates were removed. A single reviewer (N. C.) then screened titles and excluded those clearly irrelevant. The remaining citations were then uploaded to Covidence (Veritas Health Innovation, Melbourne, Australia; available at https://www.covidence.org/) to manage the screening process. Two independent reviewers screened abstracts against eligibility criteria and were excluded by agreement. Full texts were obtained and screened in the same manner. Disagreements at both stages were resolved through discussion.

### 2.6 Appraisal and cluster searching

Evidence from the main search and those identified in the background search and that met the eligibility criteria were appraised using a bespoke tool. Appraisal considered *relevance*—if the source could contribute to the development and/or testing of theory within the review; and *rigour*—if the source was coherent and trustworthy.^37^, ^38^ Articles were rated as ‘high’, ‘moderate’, ‘low’ or ‘exclude’ (see Table 2). Two reviewers independently appraised each article, ratings were compared and any discrepancies were resolved through discussion.

**Table 2:** Definitions of appraisal outcomes applied in this review

Appraisal level	Definition
High	Evidence source has high relevance to the review. The framing of the research and research questions are highly matched to the review questions. The empirical findings are clearly described and there is a rich description of the process and context. This evidence has the potential to advance the theoretical output of the review.
Moderate	Evidence source has moderate relevance to the review. The evidence reports on a related intervention that targets similar outcomes of interest or describes middle‐range theories that may inform the review. Evidence may not contribute empirical data to populate the CMOCs but includes important areas to inform thinking and development.
Low	Evidence seems relevant to the review questions and the initial programme theories, but is relatively thin in terms of description. The source may only include one idea or statement about any part of the CMOC, but it remains useful.
Exclude	Evidence source was considered promising on initial screening but, upon full‐text reading, was found to not correspond with the review questions or initial programme theory(s), or does not describe at all the context or the mechanisms (or process).

Abbreviation: CMOC, context‐mechanism‐outcome configuration.

Cluster searching through citation tracking, reference checking and database searches was used to identify evidence sources that were either directly related to the index paper or that could contribute to our theoretical or contextual understanding; Booth et al.^39^ refer to these as ‘sibling’ (direct) and ‘kinship’ (indirect) sources, respectively. Documents that were appraised as ‘high’ or ‘moderate’ were prioritized in cluster searches.

### 2.7 Data extraction

Evidence appraised as either high or moderate underwent data extraction. Full‐text articles were read and reread, and relevant information were coded and then extracted by either N. C. or T. K. A second reviewer checked coding and extraction to ensure key data were not missed. Descriptive characteristics of the included evidence were recorded in a Microsoft Excel file.

### 2.8 Data synthesis

An inductive approach was taken to support the initial coding of the data and to identify key concepts, patterns, relationships and outcomes from within the evidence. The CMOC framework was then applied to categorize codes and support ongoing comparison across the data. A lack of theoretical engagement was identified in the evidence; however, relevant substantive theories were selected and retroductively applied to the evidence to support analysis and explanation. The reviewers found it productive to start from the endpoint (the Outcome) and to work backwards to elucidate resources and responses (M—mechanisms) and key contextual (C) factors in the configuration. Preliminary CMOCs were constructed by T. K. to explain mechanisms operating at the individual (provider, receiver), service and system levels. CMOCs were shared with N. C. and C. C‐G. and reviewed until the authors were confident these adequately reflected the available evidence. Diagrams were used to illustrate relationships within and between the CMOCs and to support refinement. A programme theory was developed and iteratively updated to assess the level of explanatory fit with the CMOCs.

## 3 RESULTS

**Figure 1:**
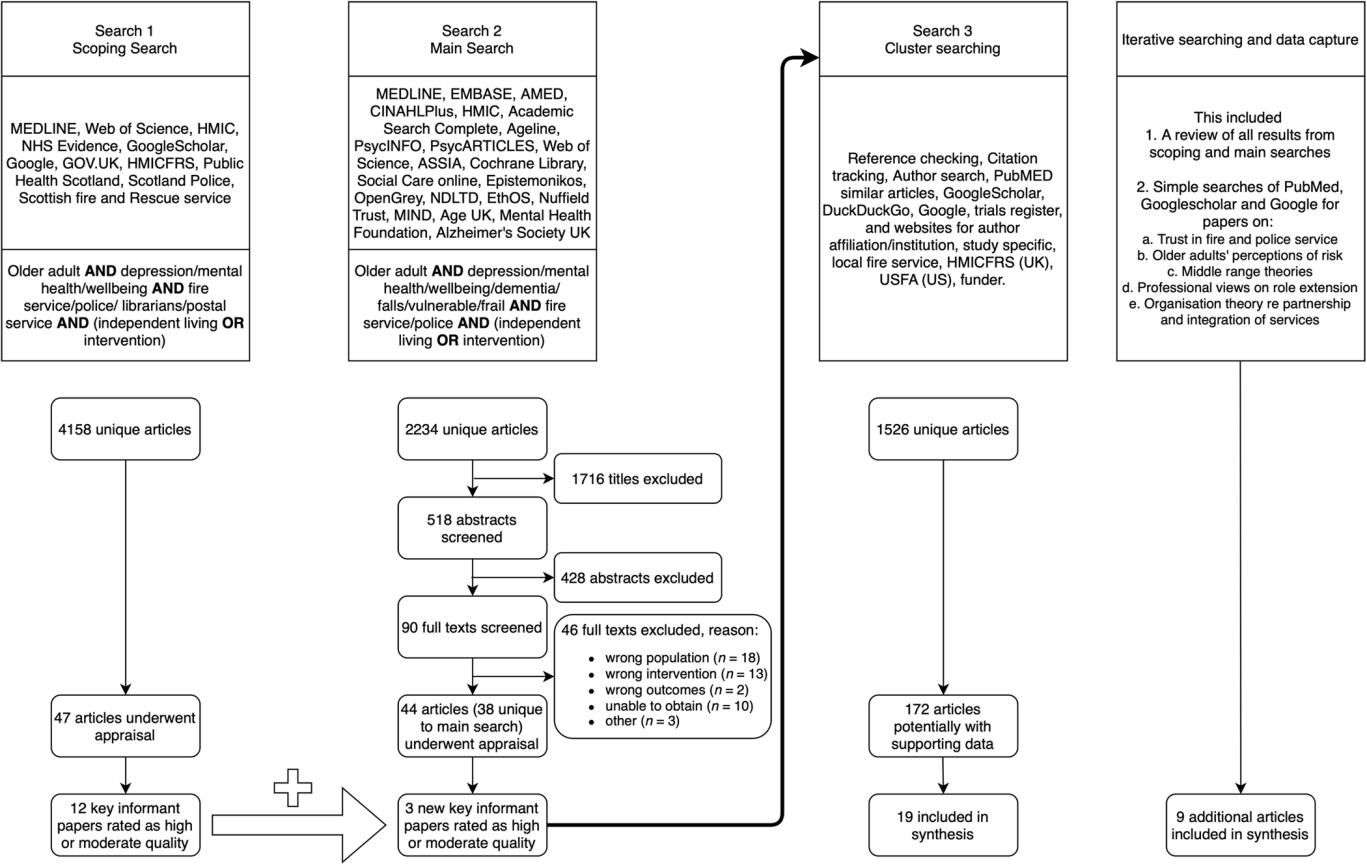
Flow diagram of literature search and retrieval stages.

**Figure 2:**
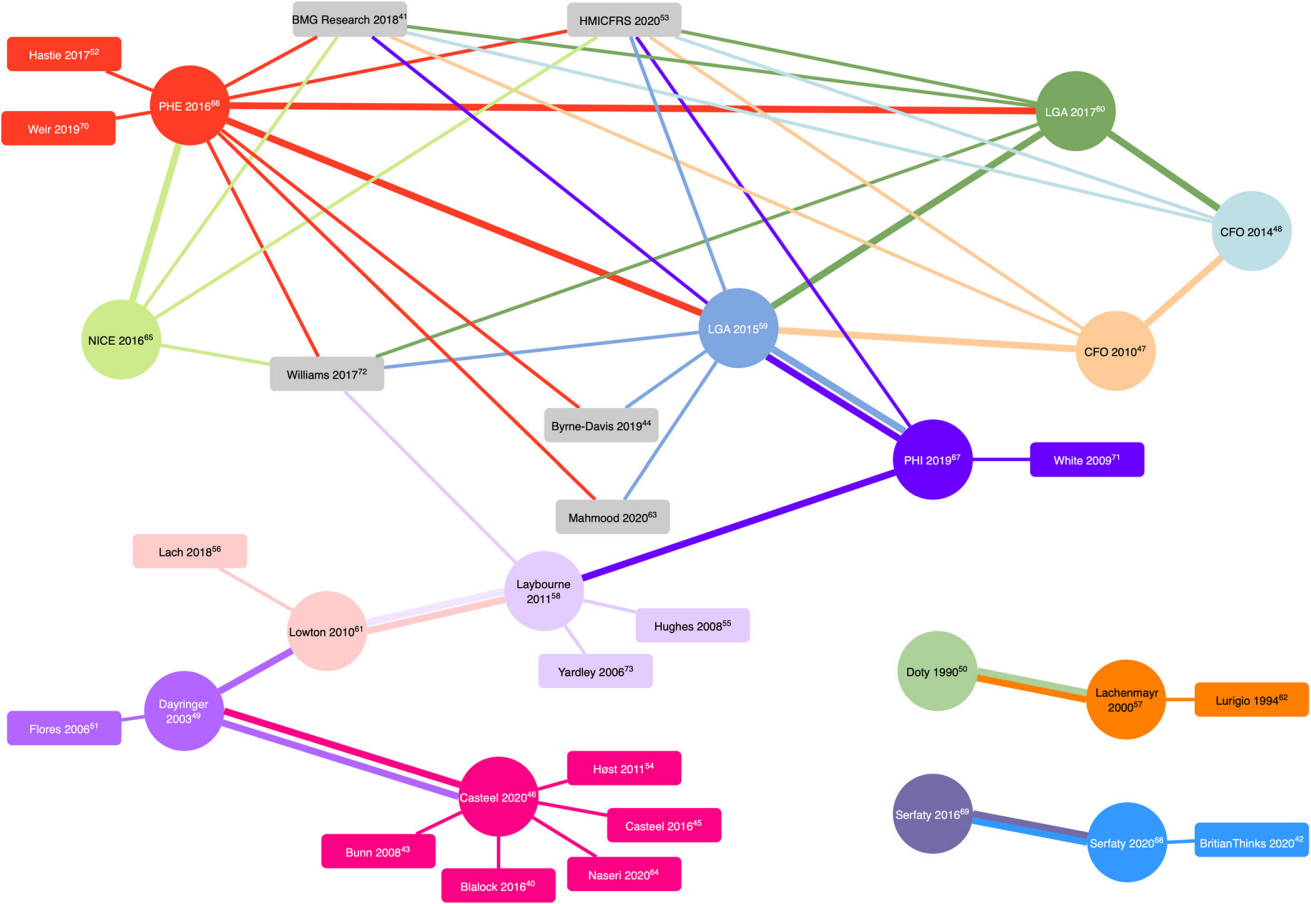
Visual representation of cluster search results. Nodes (circles) represent key informant papers, rectangles represent papers identified through cluster searching used to inform synthesis. Colour of the rectangle and line linking papers denote key informant articles that the paper was identified through, grey rectangle denotes multiple key sources.

In total, 34 documents^40^, ^41^, ^42^, ^43^, ^44^, ^45^, ^46^, ^47^, ^48^, ^49^, ^50^, ^51^, ^52^, ^53^, ^54^, ^55^, ^56^, ^57^, ^58^, ^59^, ^60^, ^61^, ^62^, ^63^, ^64^, ^65^, ^66^, ^67^, ^68^, ^69^, ^70^, ^71^, ^72^, ^73^ identified from systematic and cluster searches informed the development of CMOCs. This evidence comprised peer‐reviewed journal articles (primary research, study protocol), service level reports and evaluations, published between 1990 and 2020. These were supplemented with nine further documents^18^, ^74^, ^75^, ^76^, ^77^, ^78^, ^79^, ^80^, ^81^ obtained from iterative ad hoc searches and data capture to support refinement and articulation of the programme theory. A document flow diagram is provided to explain evidence search and retrieval processes (Figure 1). Fifteen interventions were identified in total; key characteristics of which are summarized in Table 3. Of these interventions: 10 were provided by Fire and Rescue Services or Departments and targeted falls and fire (6), dementia (1) or multiple problems (e.g., falls, frailty, social isolation, winter warmth, bowel cancer screening, alcohol consumption) (3); four were provided by the Police and targeted dementia or memory loss (2) and victims of crime (2); one intervention to identify issues related to vulnerability was delivered by both Fire and Rescue and Police. Cluster searches were conducted based on the key informant papers, as illustrated in Figure 2. Our searches identified a dearth of high‐quality research evidence; the majority of documents were identified in the ‘grey literature’ (e.g., service reports, public policy documents and evaluations).

**Table 3:** Key informant papers retained for synthesis

First author (year) Country	Study method	Setting	Intervention^a^	Middle‐range theory identified	‘Nontraditional’ provider and role^a^	Collaborators if applicable
*Fire and Police Services*						
Chief Fire Officer (2010)^47^	Case report	Home visit	Safe and Independent Living (SAIL): Community partnership to ensure referral of ‘vulnerable’ older adults (≥50 years) to appropriate services.	NR	FRS and police: Undertake usual activities plus complete an A4 referral form with clients who identify which services, support of information they want to access.	Age UK, plus 70 referral partners incl. Local Authority Social Care, Dorset Blind Association, Environmental Health and Memory Advisory Service
England						
*Fire Service*						
Casteel (2020)^46^	Service evaluation	Home visit ± group presentation	Remembering When^TM^ program: Falls and fire safety program for older adults (≥65 years).	Health Belief Model	Fire department outreach team: Provision of Remembering When program.	Home care organizations (to recruit only)
USA						
Chief Fire Officer (2014)^48^	Case report	Home visit	Hull 2020 project: Vision ‘working together better to enable the people of Hull to improve their own health and wellbeing and their aspirations for the future’. Includes falls prevention and response.	NR	FRS: Development and delivery of ‘Safe and Well checks’, and delivery of a response to falls service with partner agencies.	Led by National Health Service (NHS) Hull Clinical Commissioning Group; and including Yorkshire Ambulance Service; Humber NHS Foundation Trust; Humberside Police; Hull and East Yorkshire Hospitals NHS Trust
England						
Dayringer (2003)^49^	Action research (literature review, evidence search, interview)	Home visit, scene of the incident	Tulsa Fire Department Risk Reduction Program: Includes education, home safety assessment, home modifications and referrals, exercise, and medication review. Aims to reduce injury and loss of life in older adults from falls, suicide, traffic accidents and fires.	NR	Fire department: Home safety assessment, modifications, referral to relevant services, exercise intervention.	Potential collaborators suggested, including local community and health/social care organizations
USA						
Laybourne (2011)^58^	Overview with reference to a proof‐of‐concept study (Lowton, 2010—see below)	Home visit	Southwark and Lambeth Integrated Care Pathway for Falls. Reciprocal intervention between fire service and NHS. Fire and Rescue service conduct falls risk assessments during home fire safety checks with referral to falls clinic if required, and nursing teams at falls clinics provide fire safety information and a postcard for self‐referral to home fire safety visit from fire service.	NR	FRS: During home fire safety visits, invite older adults for a falls risk assessment; referral to falls clinic if at risk.	NHS
England						
Local Government Association (2015)^59^	Case studies	Home visit where applicable	Showcase of FRS's involvement in services addressing the public's health and well‐being: including dementia, as ‘health champions’ (wider health and wellbeing issues incl. mental health), falls prevention.	NR	FRS: Training to become ‘dementia friends’ thus improving help and support to older people with dementia; ‘health champions’ delivering basic health advice and signposting to relevant services usually delivered alongside normal fire safety checks and/or with partner agencies; identification of older adults at risk of falls and referral to local health service's falls prevention team.	Alzheimer's Society; Local council (Wigan); Age UK, local house associations, local councils; Dementia UK; North West Ambulance Service, Greater Manchester Police
England						
Local Government Association (2017)^60^	Case studies	Home visit	Showcase of FRS's involvement in services addressing the public's health and well‐being, including identifying residents at risk of (fire and) falls and referring where needed; SAIL: community partnership to ensure referral of ‘vulnerable’ older adults (≥50 years) to appropriate services; Public Health England pilot to reduce winter pressure on NHS focusing on most vulnerable groups, including FRS safe and well checks.	NR	FRS: Targeting residents at risk of (fire and) falls for assessment and where necessary referral to relevant services; extension of safe and well checks to include an A4 referral form with clients who identify which services, support of information they want to access or to include assessments, including risk to falls and if necessary, referral or signposting to other agencies.	Age UK, local authorities, and health services; multiagency, including police, NHS, welfare and councils
England						
Lowton (2010)^61^	Protocol for a proof‐of‐concept study. Mixed methods	Home visit	Collaboration between FRS and NHS to improve well‐being (falls) and safety (fire) of vulnerable older adults.	NR	FRS: During home fire safety visits, invite older adults for a falls risk assessment; referral to falls clinic if at risk.	NHS
England						
National Institute for Health & Care Excellence (2016)^65^	Brief case report	Home visit	Winter Warmth pilot.	NR	FRS: Adaptation of Safe & Well visits to include winter warmth checks (falls & frailty, warm homes and isolation) with signposting to relevant services.	Public Health England
England						
Public Health England (2016)^66^	Service evaluation	Home visit	Broadening of Safe & Well visits to include a focus on risk factors for winter‐related illnesses (pilot focused on prevention of falls, cold homes, social isolation and promotion of flu vaccination).	NR	FRS: Adaptation of Safe & Well visits to incorporate broader health issues incl. winter‐related ill‐health, including falls, cold homes, social isolation and flu. Identification of households at risk and provision of targeted interventions, that is, advice and/or referral, to mitigate risk.	Health (NHS) and Social care services: Public Health England, NHS England, Local Government Association, Age UK
England						
Public Health Institute (2019)^67^	Service evaluation: mixed methods	Home visit	Extension of FRS Safe and Well visits.	Behaviour change theory, for example COM‐B model	FRS: Extension of Safe & Well visits to include assessments and referral to specialist advice/services regarding bowel cancer screening, falls prevention, smoking and alcohol consumption.	NHS
England						
*Police Service*						
Doty (1990)^50^	Training programme evaluation (mixed methods)	n/a	‘Assisting the Elderly Who Have Serious Memory Loss’—training intervention regarding dementia for police officers. Training covers: memory loss, family issues, coping behaviour and communication skills.	NR	Police: Receive training on how to interact with older adults with dementia and familiarity with local community resources. Thus, enabling officers to provide advice and signpost/refer to other information, services and support within the community.	
USA						
Lachenmayr (2000)^57^	Training evaluation	Out‐reach	Safe Return: community‐based intervention to increase the safety of people with Alzheimer's disease.	Constructs of Diffusion Theory; Social Learning Theory	Police: Receive training regarding Alzheimer's disease, enabling the provision of outreach activities (education and enrolment in Safe Return register).	Alzheimer's Association
USA						
Serfaty (2016)^69^	Feasibility RCT using mixed methods	Home visit	Helping Aged Victims of Crime (HAVoC): Identification and referral to treatment as usual (TAU) or TAU plus CBT‐based manualized treatment (Victim Improvement Package [VIP])	Cognitive models of trauma	Police: Identification and initial screening of older victims of crime; referral to (research team for) intervention/services.	Clinical Trials Unit (University of Aberdeen); Victim Support; Accredited psychological therapist
England						
Serfaty (2020)^68^	RCT—Protocol only	Home visit	VIP: Follows HaVoC feasibility study above.	NR	Police: Identification and screening of older victims of crime for anxiety and depression. Signposting to primary care	GP; Research team; PRIMENT Clinical Trials Unit; Mind (accredited psychological therapist)
England						

Abbreviations: FRS, Fire and Rescue Service; n/a, not applicable; NHS, National Health Service; NR, not reported; RCT, randomized controlled trial.

^a^ Where multiple interventions or elements are reported, only those relevant to this review are given in this table.

### 3.1 Analytic themes

In this section, we provide a narrative summary of the coded evidence, presented as four concepts: legitimacy, focus on risk, intervention flexibility and organizational integration. See Table 4 for a summary of themes and CMOCs; see Supporting Information File S2 for CMOCs with supporting data.

**Table 4:** Summary of Context‐Mechanism‐Outcome Configurations

Theme	Subtheme	Context	Mechanism	Outcome	Example data
Legitimizing extended roles	(1) Professional status (traditional role)	Perception of public services	Trust facilitates interaction	Willing to engage at the first instance	‘Most of the beneficiaries attached a significant level of trust in the FRS and their staff to provide support through the proposed visit. A few of the beneficiaries said that they would not have been involved in the pilot had it been run by another organisation; “the fire service you can trust”. Staff carrying ID or badges reassured them that they were in safe hands’.^66^(p.28)
	(2) Acceptance of ‘Nontraditional’ role	Lack of association with health	Prior expectations support awareness	Likely to accept information	‘Respondents are most likely to think that extinguishing fires (87%) and promoting fire safety (82%) should be the responsibility of their local Fire and Rescue Service. Respondents perceive preventative and outreach activities to be a lower priority, but a majority of respondents still feel that their FRS should be responsible for them’.^41^(p.8)
		Service‐level priorities	Respected officials provide an endorsement	Likely to accept the role	‘Early indications are that there would be no shortage of applicants from the firefighter cadre to carry out the new role. It would also provide an opportunity for firefighters to rotate in and out of this specialist area enhancing their skills’.^60^(p.13)
Focusing on risk	(1) Awareness of being at risk	Receiver beliefs about risk	Empathy through experience (direct or vicarious)	Willing to acknowledge the risk to self	‘We observed that fire service personnel shared real life stories to vividly convey the risks associated with fire that led to a significant increase in risk perceptions related to fire. However, fire service personnel had fewer stories related to falls and their consequences to share with participants during the program. Providing additional resources such as DVD testimonials to those delivering the RW program may help increase the overall impact of this program’.^46^(p.725)
					*[Data indicates lack of resource to support empathy]*
		Provider beliefs about risk	Acknowledging links between risks for traditional concerns (fire/crime) and health and wellbeing concerns	Likely to promote risk reduction behaviours	‘A primary concern of law enforcement officers is the constant precaution for life‐threatening situations. They approach each situation mindful of immediate danger and their responsibility to enforce the law. After officers ensure safety, they evaluate the situation, such as an assault or felony, in terms of authority to arrest. A situation involving memory loss patients, however, should incorporate conciliatory procedures’.^50^(p.360)
	(2) Managing and mitigating risks	Staying safe at home	Shown how to identify and address hazards in the home	To enhance the sense of control over home	‘Around one in ten beneficiaries reported improvements in awareness of the risk of falls, stating that the home visits identified and addressed hazards and informed them of how to reduce the risk of falling when on the move’.^66^(p.36)
		Prioritizing risk to independence	Offering choice to promote autonomy	Reduced sense of risk to independence	‘A couple of Outreach Team members had to overcome hesitation from older adults about inviting them into their homes: “…they were a little bit skeptical at first about us coming into their homes and trying to tell them what to do but we just went there” and, “Today we're not here to judge you or we can't force you. We're just here to help you try to make things better. Keep you in your home as long as we can”. They realized that so they were good with it’.^45^(p.36)
Intervention flexibility	(1) Supporting individualization	Prior knowledge and experience	New information/activities put in context	Likely to engage in positive behaviours	‘A common characteristic of interventions that may widen socioeconomic inequalities in health appears to be “a reliance” on self‐directed behaviour change,^71^ as is the case with the Safe & Well visit. Inequalities may be introduced at different stages; such as in uptake and engagement, and in how individuals respond to an intervention. White et al.^71^ note that “the problem with ‘one‐size‐fits‐all’ interventions has been recognised” and that interventions tailored to the needs of sub‐groups within a target population may be more likely to result in outcomes that are more equitable’.^67^(p.51)
		Delivering a novel intervention	Knowledge and experience to contextualize	Likely to improve acceptability	‘A few staff reported that they found that the test was sometimes unnecessary or impractical. For example, staff reported that they were often able to establish the vulnerability of a person by observing how quickly they answered the front door and watching them move around the house, as well as when they were developing an escape route in case of fire with the beneficiary. In addition, some staff found it impractical to roll out 3 metres of measuring tape, especially in properties which were very small or had limited space’.^66^(p.33)
	(2) Harnessing support networks	Existing support network	Facilitating new or repurposing existing contacts	Likely to utilize the support	‘One elderly resident who was helped by SAIL said it had “unleashed an army of people” to see her’.^60^(p.10)
					‘We are looking at the onward acceptance of referrals as we know there is a big drop off between referral and acceptance into services particularly for falls. Therefore we are not sure the pathway is good enough at the moment and we may be missing opportunities. (Local authority, Merseyside)’.^67^(p.37)
		Home visits as social interaction	Opportunity to talk with a trusted person	Social participation	‘Nine [out of 55] participants specifically mentioned that they appreciated interacting with and learning new information from the Outreach Team members who conducted the group presentations and home visits. As one participant stated, “I really enjoyed the visit from the fireman… I thought it was very useful and he's a lot of fun, too”’.^45^(p.33) Ellipses added for context.
Organizational integration	(1) Service culture, capacity and context	Culture of teamwork	Group learning reinforces team culture/identity	Improved confidence, reduced stigma	‘Frontline staff that had face‐to‐face training were generally more positive than staff who had attended webinars’.^66^(p.25)
					‘“When accompanying fellow advocates on visits (due to lack of vehicles) and seeing how they deliver Safe & Well, my confidence has improved. Also over time getting used to the new format. My confidence has improved from regular CPD days too”. (Advocate, more than 100 Safe & Well visits made)’.^67^(p.18)
		Dedicated roles and responsibilities	Enhance credibility and prevent overburden	Unlikely to make compromises	‘One of the major barriers Outreach Teams experienced in completing the study activities, which included Remembering When™ program delivery and completing data collection instruments, was limited time and staff support. One Outreach Team member described the issue as follows: “I mean I really enjoyed it, but it was we were trying to get a lot of visits in a short amount of time. For me, being really the only person doing them, I felt a little scatterbrained [laughter] or a little hectic some days trying to make sure I was getting everything done”’.^45^(p.40)
		New intervention context	Opportunity for trial and error in the context	Likely to achieve referral targets	‘There is an indication that implementation is more effective on a smaller scale, across a single area, compared to large scale implementation across a metropolitan area, with a diverse population. Both Staffordshire and Gloucestershire delivered the pilot incrementally over a smaller geographical area than Greater Manchester and reported fewer challenges and problems in delivering the home visits. The benefits of this approach were that they could learn from problems as they went along and alter parts of the pilot without too much disruption’.^66^(p.10)
	(2) System‐level collaboration	Dynamic system	Leadership and communication establish new boundaries	Likely to establish credibility (organizational legitimacy)	‘Nearly all partner organisations indicated that the pilot had led to improved communication and relationships between themselves and the FRS and they intended to further develop joint working in the future’.^66^(p.9)
		Organizational knowledge	Opportunity to share local knowledge improves relationship	Likely to enhance reciprocity	‘Humberside FRS have played a leading role in Hull 2020, with a Group Manager leading the frail and elderly work stream and they have seconded a Station Manager full time into the CCG. This secondment is designed to improve the already strong links with the CCG, and enable HFRS and the CCG to better understand each other's work. The SM has led on a number of the collaboration projects mentioned above, and acts as a conduit and “translator” between the worlds of health and fire. His brief is to also explore what else could be provided with our other CCGs, not just Hull, and this is already starting to deliver useful outcomes’.^48^(p.2)
					‘The most effective method to reach the target population was to combine resources with a coalition of organisations who were providing services to older adults’.^49^(p.3)

### 3.2 Legitimizing extended roles

Public services are receiving increasing recognition as health and social care assets.^18^ At a strategic level, the boundaries of traditional services appear to have blurred. For people on the ground (service providers and receivers), traditional roles form the basis of interactions; legitimacy to work outside of these boundaries has not yet been established.

### 3.3 Professional status

Members of the public generally hold public services in high regard with respect grounded in perceptions of professional integrity and competence aligned to traditional roles.^41^, ^66^ For many, being able to identify an individual as a member of a respected service establishes an immediate basis for trust, which enables public service personnel a ‘foot in the door’ to interact with members of the public inside and outside of their home. However, individually held attitudes and beliefs about public services are shaped by past interactions and experiences. Fire and rescue service staff described how some older adults were sceptical about allowing them into their homes and that trust still had to be earned.^45^ People from ethnic minority backgrounds and older adults that feared being scammed also reported negative views of public services and/or of those potentially posing as representatives of these services.^52^ Thus, trust based on professional reputation seems consistent for the majority of the public but should not be assumed. From a service provider perspective, Police and Fire and Rescue Service staff recognized a high level of trust and respect among the public based on their professional status—protecting this trust was paramount among staff.^65^, ^66^

#### 3.3.1 Acceptance of ‘nontraditional’ role

Uncertainty regarding the acceptance of nontraditional roles has been reported.^53^, ^65^, ^67^, ^79^ Members of the public described a lack of awareness about health promotion roles undertaken by the Fire and Rescue Service; many did not expect to be asked questions about topics outside of traditional remit(s).^53^, ^65^, ^67^ A lack of expectation often caused surprise and/or confusion and increased potential for disengagement. Members of the public were alert to privacy and sensitive to topics they perceived to be stigmatizing and which could cause embarrassment or offence. Positive engagement seemed to be related to the attitude of the individual member of the public and the approach of the individual member of staff; the ability to establish rapport and maintain professional integrity during conversations was crucial.^67^ A communication strategy that utilizes the opportunity to raise awareness about these nontraditional roles, also seems important to establish health and social care topics as an acceptable and legitimate ground for nontraditional services.^61^, ^65^ For service providers, endorsements of new roles from reputable individuals helped to support acceptance by generating credibility in nontraditional activities and clarifying how such actions added value to the service.^78^ Fire and Rescue Service staff, in particular, welcomed new opportunities to upskill themselves^60^ and services have been active in this space.^78^

### 3.4 Focusing on risk

Risk in relation to avoiding or preventing immediate danger is of key concern to the Police and Fire and Rescue Services in their traditional emergency‐response roles. However, these attitudes towards risk in the context of physical and mental health conditions may not be mutually valid. How risk about nontraditional topics is conceptualized and communicated has important implications for engagement and intervention success.

#### 3.4.1 Awareness of being at‐risk

Older adults that are referred to, or identified by, nontraditional providers were unlikely to have self‐identified as being at risk of, for example, risk of falling. Interventions designed to reduce the risk of falls are likely to fail in circumstances where recipients either do not feel sufficiently motivated to engage (i.e., have a low sense of risk), or their lack of awareness about risk contributes to a false sense of security.^40^ For example, older adults often rate susceptibility of experiencing a fall as low (particularly men), despite incidence data to the contrary, and seek to avoid being perceived as potentially frail.^43^, ^45^, ^54^, ^55^, ^58^, ^67^ Lack of engagement with falls advice may be due more to the threat to identity and independence, rather than ignorance.^73^ Thus, interventions that promote risk awareness, empathy and a sense of personal capability and control may better enable older adults to recognize the relevance of an intervention and improve motivation to take action.^52^, ^55^ The use of vicarious experiences has been demonstrated to support empathy among recipients in the context of fire prevention (e.g., video resources involving house fire case studies), but not aspects of health and wellbeing.^46^ Assuming a broader focus on the quality of life could also be beneficial to avoid stigma.^67^

From a provider perspective, beliefs about risk among police and fire and rescue service personnel may focus on danger avoidance; this reinforces identities embedded in traditional emergency‐response roles. However, in the context of health and wellbeing more conciliatory approaches that support empathy may be needed to address public needs, as highlighted in circumstances of dementia.^50^

#### 3.4.2 Managing and mitigating risks

Older adults reported safety in the home and maintaining independence as two key motivators for behaviour change in the context of falls prevention.^45^

Interventions that support older adults to identify hazards (e.g., trip hazards) in the home and offer straightforward, practical solutions to improve safety seem more acceptable. Having the opportunity to practice positive behaviours enhanced competence and confidence, but crucially control over their home environment.^45^, ^46^ Older adults described scepticism and hesitation regarding interventions delivered in the home, as these invited unwanted judgements (from public services, neighbours), which threatened their independence posing a barrier to engagement.^52^, ^73^ To address these concerns, service providers gave verbal reassurance to older adults that interventions were designed to prolong, not threaten, independence and personnel would be nonjudgemental—this approach seemed to overcome initial hesitancy.^45^ Testimonials from older adults that had previously engaged with the intervention helped to support this stance.^45^ Intervention resources that promote individual capacity and enablement may support motivation to engage as they act to reinforce independence. However, this approach was often lacking in the existing interventions. Interventions that rely solely on distributing information may not be sufficient, as this places the burden on individuals to be proactive in seeking help.^68^

### 3.5 Intervention flexibility

Delivery with fidelity is often a key concern when implementing any novel intervention. In the context of nontraditional services, where providers are establishing their confidence and may not possess the awareness and/or knowledge of health and social care, delivering an intervention‐as‐designed was often prioritized. A lack of flexibility in training and intervention design may limit applicability and damage confidence.

#### 3.5.1 Supporting individualization

One‐size‐fits‐all interventions were considered to be problematic as they did not adequately account for individual experiences, existing behaviours and social inequalities.^67^, ^71^ Intervention providers described contextual barriers and challenges when delivering interventions as described. Practical problems were described when conducting assessments in a person's home, such as mobility tests as part of falls prevention interventions, due to lack of space.^67^ Providers did not seem to anticipate recipients would already be engaged in positive behaviours; negative examples of ‘nit‐picking’ were described, which may undermine credibility and trust.^45^ Risk assessments provided a useful means of identifying otherwise hidden health and social needs and provided a prompt to discuss wider issues and experiences.^48^, ^59^, ^60^ Interventions commonly lacked opportunities to identify existing strengths of older adults, such as behaviours, resources and networks to build these into action planning. Opportunities for more than one point of contact with a member of the public were highlighted as key for rapport and behaviour change.^63^ Training delivered to providers often failed to cover complex communication strategies (or skills) or build in opportunities for reflective practice.^44^, ^63^

#### 3.5.2 Building on support systems

Older adults are commonly described as relying upon existing support networks, such as friends and family, rather than accessing support services for health and wellbeing concerns.^45^, ^69^ Police and Fire and Rescue Services recognized interactions with older adults as opportunities to sign‐post (i.e., provide direction) to partner organizations to enhance individual support networks.^60^ However, the acceptance and uptake of onward referrals to external services varied between recipients and interventions. Older adults reported declining suggested referrals as they felt they were already accessing appropriate help and support; this may also link to concerns about losing independence.^67^ Large drop‐offs between referral and acceptance into services were also reported, suggesting referrals that were being made may have been inappropriate (i.e., did not meet specialist service eligibility criteria).^67^ This is problematic for older adults, as they were denied opportunities to expand their support network, and intervention effectiveness, as many interventions used the number of referrals as a short‐term indicator of success without assessing the long‐term outcome of the referral.^66^, ^67^

The opportunity for nontraditional providers to offer social and emotional support to older adults should not be overlooked, as routine social interactions may complement the existing coping strategies of older adults.^45^, ^69^ Social support was described as an unintended consequence of three interventions^45^, ^46^, ^67^ and could inform future outcome measures and/or mechanisms for change.

### 3.6 Organizational integration

The successful implementation of interventions delivered by nontraditional providers depended to a large extent upon appropriate integration at a service level and health and social care system level. Culture, capacity, context and collaboration were key underpinning elements of successful interventions.

#### 3.6.1 Service culture, capacity and context

Delivery of training provided a useful opportunity to reinforce credibility and cultural values in the context of nontraditional roles. Group learning with others through face‐to‐face training, rather than online learning, role playing and shadowing improved confidence and preparedness.^50^, ^57^, ^66^, ^67^ Group learning reinforced identities linked to teamwork.^72^ Face‐to‐face training delivered by respected individuals or groups was also more effective.^57^

A lack of sufficient time and staff support to conduct nontraditional interventions were identified as a key barrier.^46^ Clarity at the outset about the scale of the role, having dedicated staff to deliver the intervention, and experience of delivering similar interventions helped to prevent staff burnout and ensured the quality of delivery.^46^, ^49^, ^66^

Implementation on a small scale, followed by incremental spread, seemed to support success and avoid widespread disruption by enabling contextualization, learning through trial and error and facilitating dialogue between relevant stakeholders.^44^, ^66^

#### 3.6.2 System‐wide collaboration

Collaborating partners described improved communication as a result of intervention roll‐out and established grounds for future work; however, engagement during planning stages was optimal but often lacking.^66^ Preintervention Effective collaboration facilitated the sharing of resources, including people, referral processes, local and specialist knowledge, and shared intervention messages.^47^, ^49^, ^61^, ^65^, ^66^ Having established referral pathways with awareness about eligibility criteria for service entry was critical.^66^, ^67^ Selection of an appropriate stakeholder to link nontraditional and traditional services was advantageous; in some instances, secondment roles were utilized.^48^ The wider recognition of the Fire and Rescue Service as a health asset, through the publication of the consensus statement (NHS England, Public Health England, the Chief Fire Officers' Association and Age UK) provided context and impetus for collaboration.^65^, ^66^

### 3.7 Incorporating substantive theory into the programme model

**Figure 3:**
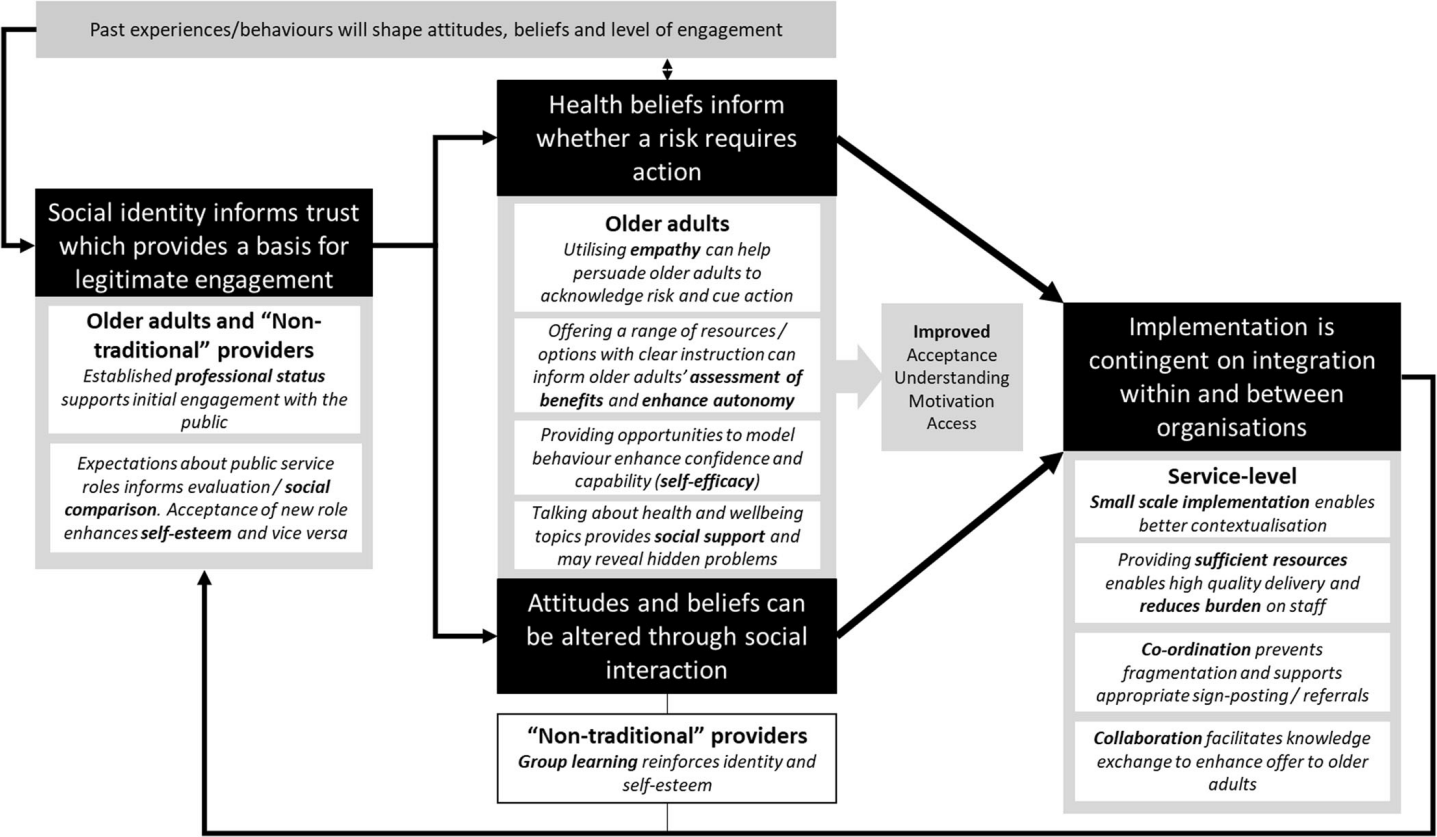
Final programme model incorporating substantive theory.

The use of theory to inform interventions included in this study was rarely made explicit. Thus, through retroduction, relevant theories were identified to enhance our understanding of the key mechanisms and inform the development of the final programme theory (Figure 3).

Professional status provides the opportunity for interaction between public services and members of the public.^41^ Social Identity Theory^81^ posits that individuals will self‐categorize themselves and others as belonging to certain groups based on salient factors (e.g., similarities within a given context), which they continue to evaluate through social comparison—this assessment informs an individual's sense of their social identity and self‐esteem (e.g., level of positive association with a particular group). Though not explicitly described in the evidence, Social Identity Theory offers a lens through which to understand the role of professional identities in the context of nontraditional activities. Police and Fire and Rescue Service personnel have strongly held identities based on traditional roles, values and activities that are perceived as congruent and inform social expectations. New roles and activities may therefore appear incongruent, which can lead to resistance by service providers and receivers.^74^ Improving congruence between traditional and nontraditional roles and identities, among service providers and recipients, offers a key mechanism to support acceptance and engagement. For example, for Fire and Rescue Service providers improving awareness about the association between fire risk and mental health may enhance correspondence between roles.

Perceptions of being at risk were identified as an important mechanism to support engagement by older adults and conciliatory actions among providers. Social Identity Theory helps us to understand how, through social comparison, older adults may be reluctant to associate themselves with identities that carry stigma and negative connotations, such as frailty, vulnerability and mental ill‐health. However, the *Remembering When* intervention demonstrated how utilizing vicarious experience and empathy can promote within‐group identities associated with being at risk of falls.^45^, ^46^ The Health Belief Model^77^ was also described in some evidence and is relevant here.^45^, ^46^ The model includes six key components: perceived susceptibility, perceived severity, perceived benefits, perceived barriers, cue for action and self‐efficacy. This model helped to further delineate underpinning mechanisms and chains of causality. Self‐efficacy was described or implied, in the absence of the Health Belief Model, in relation to motivation for engagement and behaviour change^64^, ^65^ and learning.^57^ Self‐efficacy refers to judgments about one's own capability to execute a course of action; elements of the theory include: mastery (through experience), vicarious experience, social persuasion and physiological cues.^82^ This has relevance to older adults as a determining factor in whether new behaviours, introduced through nontraditional interventions, are enacted. Efficacy can also be assessed at a group level, referred to as collective efficacy; this has potential relevance at the organizational level when implementing a new intervention and also acts as a key component of Social Learning Theory.^76^ Interventions in the current study were successful where older adults identified with being at risk, could recognize relevant risk behaviours, and were empowered to take control of their situation (e.g., mastery of their home environment)—often through practical strategies (e.g., removing trip hazards in relation to falls).

The implementation of nontraditional interventions and long‐term outcomes are largely dependent upon the integration of the provider within the existing, traditional, network of other health and social care providers (e.g., NHS, social care, third sector). Contingency theory helps us to understand this complex web of organizational relationships, functions and hierarchies.^80^ According to contingency theory, differentiation occurs *within* organizations at structural and functional levels (i.e., different departments within an organization assume different roles and orientations, e.g., fire fighter crews, community outreach teams), and *between* organizations (police, fire and rescue service, health and social care, third sector). Where multiple organizations are operating in the same space (as they are in the context of nontraditional providers here), integration is essential to avoid fragmentation between the different departments and services within and between organizations in terms of responsibilities, service gaps, duplication and inconsistencies.^75^ Interventions described in the current study were successful where integration was based on collaboration and co‐ordination, accomplished through voluntary agreements and mutual adjustments—evidenced by data sharing agreements, referral pathways, consensus statements and communication.

## 4 DISCUSSION

### 4.1 Summary of findings

The first objective of this realist review was to describe the evidence reporting interventions that identify and manage depression delivered by nontraditional services in the public sector, to older people. Our systematic search identified a distinct lack of evidence in the specific area of mental health. The scope of the review was adapted to include a broader range of health and wellbeing interventions; this returned sufficient evidence to support meaningful comparison, synthesis and transposition. The review retrieved intervention evidence for falls prevention, alcohol consumption, smoking cessation, social isolation (Fire and Rescue Services), dementia (Fire and Rescue Service and Police Force) and mental health crisis management (Police Force).

The second objective of this review was to develop an understanding of the mechanisms to explain how and why interventions worked. A range of CMOCs have been presented along with a programme theory model, which could be transposed and applied within a mental health context. Key conceptual themes were apparent: legitimizing expanded roles, focusing on risk, intervention flexibility and organizational integration. These themes demonstrate the complexity of the interventions and describe interactions at various ecological levels—individual, service and system levels. Through engagement with theoretical literature, which was either stated explicitly in the original evidence or delineated retrospectively, themes were further refined to construct a chain of causation and influence (Figure 3). The chain incorporates key theory on social identity (to consider the role, value and boundaries of trust and respect for public service personnel), health beliefs (of the receiver and whether specific conditions and/or topics are viewed as relevant and therefore worth taking action against), social learning (between receiver–provider, provider–provider, provider–service to rationalize the nontraditional role) and organizational contingency and collaboration (at a service‐service level and the process and relationships that either underpin or undermine an intervention).

### 4.2 Comparison with existing literature

This study adds to existing evidence for interventions designed to extend the role of nonspecialists for depression. Comparisons can be drawn against evidence related to barriers and facilitators to extended roles of providers in other settings, such as community pharmacies, lay health workers and social prescribers in primary care—though evidence for identifying and managing depression among older adults is limited. Having limited capacity due to existing roles, low awareness among the public about extended roles, interprofessional role boundaries, lack of an established referral system, lack of adequate mental health training and concern among recipients about confidentiality have been reported as barriers.^21^, ^22^, ^23^, ^24^, ^25^, ^26^, ^27^ The use of appropriate resources (e.g., information leaflets), motivated providers, providers perceived by recipients as local and trustworthy and working in collaboration with healthcare professionals have been described as important facilitators.^25^ The current review elaborates on the underpinning causal mechanisms and intervention resources that address these barriers and inform interventions for depression among older adults led by specific nontraditional providers (Police, Fire and Rescue Service).

The causal mechanisms underpinning extended home fire prevention visits conducted by the Fire and Rescue Service have been described elsewhere.^67^ The authors described a set of three programme theory statements, these focused on how the impact of awareness training on staff, public trust enabled open discussion about health and wellbeing with members of the public, and home visits as opportunities to connect members of the public with health and wellbeing services. These statements were limited in number and scope; the current review extends our understanding of such complex multilevel interventions.

As a relatively new phenomenon, social prescribing faces challenges of legitimacy in a similar way to public services working outside their traditional roles—as presented in the case of Fire and Rescue Services and Police Forces. However, the lack of an established reputation in health and social care can also be advantageous. A realist review on how social prescribing schemes link members of the public to local assets identified two overarching concepts of: creating and sustaining ‘buy‐in’ and establishing and maintaining connections.^26^ Tierney et al.^26^ described a perception of neutrality held by patients about link workers; this enabled discussion of potentially stigmatizing topics during interactions with the service, as perceived risks to autonomy and of being prescribed unwanted medications were reduced. Evidence in the current review reported similar experiences for fire and rescue service and police staff; however, limits of trust were described in relation to stigmatized topics (e.g., alcohol consumption, frailty) and negative past experiences of interactions with these services in their traditional roles.

Systematic review evidence for social prescribing identified facilitators that support causal mechanisms described in the current review: phased roll‐out of social prescribing interventions to enable adaptation, flexibility at a service level—as opposed to intervention level, as we describe—to support collaboration, mutual understanding and trust between partners preferably formalized in a partnership agreement, and a shared understanding about who to refer and how.^27^ Pensheny et al.^27^ further highlight the need for publicity campaigns to inform health and social care stakeholders and potential recipients—which has clear implications for social identity and reflects evidence in the review.^61^ Acceptance of social prescriptions is considered more likely where such prescriptions are believed by the service user to be of benefit, to match their needs and expectations, and where opportunities to disclose and address concerns were afforded to reduce stigma and enhance confidence.^26^, ^27^ These factors reflect the Health Belief Model, as utilized in the current review, particularly perception of risk (or need) and resulting cost–benefit assessment. The Health Belief Model^77^ has been incorporated into the final programme theory, building on its use in some of the review evidence. It should be noted that the effectiveness of this model in supporting behaviour change has come under scrutiny.^83^ Further evidence and testing are needed to support our understanding in both the fields of mental health among older adults and interventions delivered by nontraditional providers.

A realist review of pharmacist‐led smoking cessation support described five mechanisms related to pharmacist identity, capability, motivation, clinician confidence and public trust.^84^ Provider identity, capability (linked to adequate training), motivation and public trust resonate strongly with the current review and were typically interrelated, for example, nontraditional staff were motivated to participate where new areas of work were considered to reinforce professional and organizational identities. A lack of evidence limited our analysis of clinician views about nontraditional providers; no evidence was available in relation to mental health roles and interventions. Teamwork, integration and close working relationships are reported to facilitate the expansion of community pharmacist roles in general practice.^85^, ^86^ Evidence is needed to understand acceptability among clinicians about nonclinicians working with older adults with mental health problems; concerns have been raised previously in the context of third sector workers.^87^

### 4.3 Strengths, limitations and future research directions

This is the first realist review of the evidence for Fire and Rescue Services and Police Forces (as nontraditional providers) about their role in mental health problem identification and management for older adults. The systematic search of the literature and subsequent cluster searches revealed a distinct lack of high‐quality evidence and methodological rigour. Thus, the original scope of the review was changed following the initial systematic search of the literature to support the transposition of findings from other health and wellbeing‐focused interventions to mental health—the review shifted from what *does* work to what *could* work in this context. Interventions described in the evidence were found to be largely under‐theorized—taking a retroductive approach was helpful in this regard but limited the extent to which programme theories could be tested fully. Intervention types and settings also varied in the presented evidence; in some instances, this limited direct comparisons in the data but all evidence contributed to the programme theory. Findings should therefore be considered preliminary—further empirical evidence is needed.

## 5 CONCLUSIONS

Findings highlight the challenges and opportunities for the Fire and Rescue Service and Police Force, as nontraditional providers, to deliver interventions to identify and manage depression among older adults. The programme theory provides insight into what *could* work, how, for whom and also by whom (i.e., which public service). Evidence in the context of interventions targeting wider health and wellbeing concerns, although lacking in methodological rigour, demonstrated promising outcomes. The balance of evidence in this research would seem to suggest that Fire and Rescue Services may be better placed, due to existing community outreach activities and growing reputation and impetus for public health initiatives, than Police Forces to deliver low‐level interventions for depression. Whether it is the Police or Fire and Rescue Service, close working with partner agencies is crucial to promote the role of the nontraditional provider, build trust and credibility across the system and refine referral processes to ensure eligibility thresholds do not become a barrier; this can lead to demotivation for members of the public and nontraditional providers. Further empirical research is recommended to adequately test such interventions and to understand acceptability around such departures into mental health by the Police and Fire and Rescue Service as nontraditional providers of mental health care.

## AUTHOR CONTRIBUTIONS

All authors (Tom Kingstone, Carolyn A. Chew‐Graham, Nadia Corp) contributed to the original conception of the work. Tom Kingstone and Nadia Corp completed literature searches, appraisal and extraction. Tom Kingstone led the synthesis with oversight from Carolyn A. Chew‐Graham. All authors contributed to the drafting of this manuscript and agreed to the final version.

## CONFLICT OF INTEREST

Two authors have working roles with Health Expectations Journal (Carolyn A. Chew‐Graham: Editor‐in‐Chief, Tom Kingstone: Editorial Board member).

## Supporting information

Supplementary file 1. MEDLINE search strategies.

Supplementary file 2. Full list of CMOCs with supporting evidence.

## Data Availability

Data are available in article by Supporting Information Material. Please note this is a review article so no new data was generated.
