# Supplementary material for: Interventions to identify and manage depression delivered by ‘nontraditional’ providers to community‐dwelling older adults: A realist review
Source: Health Expect. 2022 Sep 6;25(6):2658–79. doi: 10.1111/hex.13594 (PMC9700136; doi:10.1111/hex.13594)
Supplement: Supplementary file 1 — MEDLINE search strategies. [file HEX-25--s001.docx]

# Supplemental file 1: MEDLINE (OvidSP) Search Strategies

The following table is an explanation of the symbols used in the search strategy below.

/ indicates an index term (MeSH heading)

exp before an index term indicates that all subheading were selected

.ti,ab,kf. indicates a search for a term in title/abstract/word(s) in keyword

* at the end of a term indicates that this term has been truncated.

# indicates the presence of a single character

adj indicates a search for two terms where they appear adjacent to each another

adj*n*  indicates a search for two terms where they appear within *n* words of each another

## Appendix 1a: Background/scoping search

| 1 | Aging/ |
| --- | --- |
| 2 | exp Aged/ |
| 3 | elder*.ti,ab,kf. |
| 4 | (older adj2 (adult* or individual* or people or person)).ti,ab,kf. |
| 5 | aged.ti,ab,kf. |
| 6 | 1 or 2 or 3 or 4 or 5 |
| 7 | Depression/ |
| 8 | exp Depressive Disorder/ |
| 9 | Mental Health/ |
| 10 | depress*.ti,ab,kf. |
| 11 | (mental adj2 health).ti,ab,kf. |
| 12 | (well being or wellbeing).ti,ab,kf. |
| 13 | or/7-12 |
| 14 | firefighters/ or police/ |
| 15 | Librarians/ or Postal Service/ |
| 16 | police*.ti,ab,kf. |
| 17 | fire.ti,ab,kf. |
| 18 | (firefighter* or firebrigade* or firem*).ti,ab,kf. |
| 19 | ((postal or mail) adj3 (service* or worker*)).ti,ab,kf. |
| 20 | ((post or mail) adj3 (wom#n or office*)).ti,ab,kf. |
| 21 | postm#n.ti,ab,kf. |
| 22 | mailm#n.ti,ab,kf. |
| 23 | (librari* or library staff).ti,ab,kf. |
| 24 | or/14-22 |
| 25 | Independent Living/ |
| 26 | (independent* adj2 (dwell* or liv*)).ti,ab,kf. |
| 27 | program*.ti,ab,kf. |
| 28 | intervention*.ti,ab,kf. |
| 29 | communit*.ti,ab,kf. |
| 30 | or/25-29 |
| **31** | **6 and 13 and 24 and 30** |

## Appendix 1b: Main search

| \| 1 \| exp Aging/ \| \| --- \| --- \| \| 2 \| exp Aged/ \| \| 3 \| elder*.ti,ab,kf. \| \| 4 \| (old* adj2 (adult* or individual* or people or person or man or woman or men or women)).ti,ab,kf. \| \| 5 \| aged.ti,ab,kf. \| \| 6 \| very old.ti,ab,kf. \| \| 7 \| old age.ti,ab,kf. \| \| 8 \| (late* adj (life or lives)).ti,ab,kf. \| \| 9 \| geriatric*.ti,ab,kf. \| \| 10 \| senior*.ti,ab,kf. \| \| 11 \| (retired or retiree*).ti,ab,kf. \| \| 12 \| pensioner*.ti,ab,kf. \| \| 13 \| veteran*.ti,ab,kf. \| \| 14 \| or/1-13 [older adults >60 years old] \| \| 15 \| Depression/ \| \| 16 \| exp Depressive Disorder/ \| \| 17 \| Mental Health/ \| \| 18 \| Psychological Distress/ \| \| 19 \| depress*.ti,ab,kf. \| \| 20 \| distress*.ti,ab,kf. \| \| 21 \| "low mood".ti,ab,kf. \| \| 22 \| (mental adj2 health).ti,ab,kf. \| \| 23 \| (well being or wellbeing).ti,ab,kf. \| \| 24 \| exp Dementia/ \| \| 25 \| dementia*.ti,ab,kf. \| \| 26 \| Alzheimer*.ti,ab,kf. \| \| 27 \| Accidental Falls/ \| \| 28 \| (fall or falls or falling).ti,ab,kf. \| \| 29 \| Vulnerable Populations/ \| \| 30 \| vulnerab*.ti,ab,kf. \| \| 31 \| Frailty/ \| \| 32 \| frail*.ti,ab,kf. \| \| 33 \| or/15-32 \| \| 34 \| firefighters/ or police/ \| \| 35 \| police*.ti,ab,kf. \| \| 36 \| fire.ti,ab,kf. \| \| 37 \| (firefighter* or firebrigade* or firem*).ti,ab,kf. \| \| 38 \| or/34-37 \| \| 39 \| Independent Living/ \| \| 40 \| independent*.ti,ab,kf. \| \| 41 \| program*.ti,ab,kf. \| \| 42 \| intervention*.ti,ab,kf. \| \| 43 \| project*.ti,ab,kf. \| \| 44 \| communit*.ti,ab,kf. \| \| 45 \| (liv* adj3 (home* or house*)).ti,ab,kf. \| \| 46 \| (own adj (home* or house*)).ti,ab,kf. \| \| 47 \| (household* or house hold*).ti,ab,kf. \| \| 48 \| dwell*.ti,ab,kf. \| \| 49 \| or/39-48 \| \| **50** \| **14 and 33 and 38 and 49** \| |
| --- | --- | --- | --- | --- | --- | --- | --- | --- | --- | --- | --- | --- | --- | --- | --- | --- | --- | --- | --- | --- | --- | --- | --- | --- | --- | --- | --- | --- | --- | --- | --- | --- | --- | --- | --- | --- | --- | --- | --- | --- | --- | --- | --- | --- | --- | --- | --- | --- | --- | --- | --- | --- | --- | --- | --- | --- | --- | --- | --- | --- | --- | --- | --- | --- | --- | --- | --- | --- | --- | --- | --- | --- | --- | --- | --- | --- | --- | --- | --- | --- | --- | --- | --- | --- | --- | --- | --- | --- | --- | --- | --- | --- | --- | --- | --- | --- | --- | --- | --- | --- |
