# Supplementary material for: Interventions to identify and manage depression delivered by ‘nontraditional’ providers to community‐dwelling older adults: A realist review
Source: Health Expect. 2022 Sep 6;25(6):2658–79. doi: 10.1111/hex.13594 (PMC9700136; doi:10.1111/hex.13594)
Supplement: Supplementary file 2 — Full list of CMOCs with supporting evidence. [file HEX-25--s002.docx]

Supplementary file 2. Full list of CMOCs with supporting evidence

# LEGITIMISING EXTENDED ROLES

## Professional status (traditional role)

1. **Older adults respect police and fire and rescue services (C). Being approached by a legitimate member of the service (uniforms, ID badge, vehicle) (resource) encourages older adults to engage and interact because they will feel what is being shared with them is trustworthy (response) (M). Older adults granted service representatives permission to access their home (O).**

‘We know that our brand and the esteem in which the service is held gives us access to people’s homes that others cannot achieve; people seem more likely to engage in difficult conversations with our staff than others.’ **^65^**

‘People seem more likely to engage in difficult conversations with [FRS] staff than with others.’ **^65^**

‘They believed that canvassing households they had identified within a particular area, covering them street by street, worked well to engage people because they trust and respect the FRS brand.’ **^66 (p.27)^**

'Most of the beneficiaries attached a significant level of trust in the FRS and their staff to provide support through the proposed visit. A few of the beneficiaries said that they would not have been involved in the pilot had it been run by another organisation; ‘the fire service you can trust’. Staff carrying ID or badges reassured them that they were in safe hands.' **^66 (p.28)^**

‘Most respondents are confident that their local FRS provides an effective service (86%). Reasons given for this confidence include perceptions that the FRS does a good job, even if they have not had any experience of their FRS, perceptions that response times are quick and having a trust in the FRS.’ **^41 (p.6)^**

‘An additional fear for some in engaging with public services was highlighted by Connie. She drew a distinction between the perceptions of younger people and those of older people, suggesting that for the latter there was often a fear of becoming the victim of a crime: "And I think the older people are probably fearful of being taken in, you know. You hear so many cons and schemes and scams and things like that, and they've become more, probably, isolated because of that, because they're frightened. People they would have opened the door to they’re frightened to”.’ **^52(p.166)^**

‘Clarissa, an African woman working with a community development organisation largely focused on Black African and Black Caribbean clients, suggested that for many Africans contact with authority and public services was something that had only negative connotations.’ **^52(p.167)^**

## Acceptance of “Non-traditional” role

1. **Older adults do not associate the Fire and Rescue Service with health roles (C). FRS staff making inquiries about health-related topics that members of the public were not expecting or that are considered stigmatising (negative resource), may be perceived as intrusive and/or confusing (response) (M). Older adults are likely to disengage with intervention regarding aspects of health and wellbeing (O).**

‘Respondents are most likely to think that extinguishing fires (87%) and promoting fire safety (82%) should be the responsibility of their local Fire and Rescue Service. Respondents perceive preventative and outreach activities to be a lower priority, but a majority of respondents still feel that their FRS should be responsible for them.’ **^41(p.8)^**

‘Health screening/detection is the element that respondents are least likely to say should be part of fire safety checks in homes (9%).’ **^53(p.25)^**

‘We accept that some people will not expect us to discuss health and wellbeing issues. Our internal contact centre advises callers of our safe & well & winter warmth offer at the time of booking, and we are also in the process of reviewing and updating our leaflets and resources to reflect this.’ **^65^**

‘Our evaluation found that householders were willing to engage in conversations with FRS about their health and wellbeing, supporting the central premise of the Safe & Well visit that it provides access to people that other public agencies may find difficult to reach. However, our findings also suggest that householders may be less keen to engage in conversations about their smoking and alcohol use, behaviours that they may perceive to be stigmatised.’ **^67(p.51)^**

‘Some of the responses from householders suggests that there may be boundaries for some householders in their acceptance of FRS asking health questions; they rationalise that the topics of the Safe & Well visit are ‘about safety in the home’. This suggests an acceptance of the role of FRS in fire prevention but perhaps, as yet, a lack of understanding (but not necessarily a lack of acceptance) of the extended remit of FRS to engage with the community about health and wellbeing.’ **^67(p.47)^**

‘“Depends on the person you are delivering it to, some people find it as poking our nose in the business other people don't mind so much, I've not come across anyone that hasn't got no issue with it.” (Firefighter, 10-50 Safe & Well visits)’ **^67(p.19)^**

‘Engaged with many households identified/targeted for home visit; little resistance to FRS staff undertaking the visit; fire/home safety clearly a selling point’. **^66 (p.22)^**

‘The Brigade and the fire brigade Borough Commanders work with pan-London and local agencies to identify and access individual older people who may be vulnerable. This has previously been achieved via arrangements with, for example, Age Concern, Help the Aged (now joined together as Age UK), housing associations, primary medical care centres, Local Authority departments, mobility scooter shops, and bingo halls. Other social marketing techniques such as advertising aimed at older people on television and radio has also proved effective, as has advertising the Home Fire Safety Visit service on pharmacy bags. Additionally, the Visit scheme is advertised on the Brigade’s fire engines, web site and in parts of the community where the most vulnerable people or their carers may visit, for example doctors’ surgeries, chemists, and libraries.’ **^61(p.4)^**

1. **Service-level priorities aligned with traditional roles and values (C). If highly respected officials are seen to endorse new approach, making link between traditional and non-traditional explicit, then service providers will acknowledge relevance and value (M). Staff will be more likely to support adoption into practice.**

‘Safe & Well visits have been introduced into an existing setting, that of the FRS conducting Home Fire Safety Visits. This has happened against a background shift in organisational procedures and priorities in the FRS, and work practices towards fire prevention, and greater involvement in community engagement activities.’ **^67(p.47)^**

‘When FRS began to engage both broadly and proactively with discourses of safety, relative risk and vulnerability and were compelled to change work practices, space was created allowing them to operate in non-traditional and innovative ways to deliver public sector services… this space has been used creatively… to engage in a range of activities that 15 years ago would have been considered beyond the remit of a fire brigade.’ **^78(p.220)^**

‘Early indications are that there would be no shortage of applicants from the firefighter cadre to carry out the new role. It would also provide an opportunity for firefighters to rotate in and out of this specialist area enhancing their skills’. **^60(p.13)^**

# FOCUSING ON RISK

## Awareness of risk

- 1. **Older adults not declaring to be "at risk" (C). If older adults do not experience (directly or indirectly), or cannot be persuaded to acknowledge that, 'situation X' presents a viable risk (negative resource), then they will not be meaningfully engage with risk-related information through lack of empathy (response) (M). Older adults continued to perceive themselves as not at risk (O).**

‘It seems clear that most of the people in our study were only too well aware of their likelihood of falling, but nevertheless refused to accept that they should be defined as, or behave as, potential ‘fallers’. In doing so, they explicitly rejected a number of unwelcome connotations of the concept of ‘falls prevention advice.’ **^73(p.515)^**

‘On average, the 110 participants reported very low levels of perceived susceptibility of experiencing a fall (0.85 indicating an average response between “not at all likely” and “a little likely”), perceived severity of a fall (1.56 indicating an average response between “a little serious” and “somewhat serious”), and fear of falling (0.99 indicating an average response of “a little fearful”) at baseline. The levels of these three types of perceptions about falls did not change significantly after participation in the program (Table 8).’ **^45(p.29)^**

'We observed that fire service personnel shared real life stories to vividly convey the risks associated with fire that led to a significant increase in risk perceptions related to fire. However, fire service personnel had fewer stories related to falls and their consequences to share with participants during the program. Providing additional resources such as DVD testimonials to those delivering the RW program may help increase the overall impact of this program.' **^46(p.725)^**

‘It may be that householders consider the seriousness of the risks associated with fire safety differently to those that affect their health and wellbeing. The potential for the visits to improve quality of life was recognised in the householder responses to the survey and householders appeared to understand the preventative nature of the approach. However, as Laybourne et al. (2011) report in relation to falls prevention, older people consider that “it is others who are frail and might fall”. That is householders may perceive there to be benefits of the Safe & Well visit for ‘others’ but perhaps not directly for themselves.’ **^67(p.47)^**

‘Shared findings were that many see ‘homehealth checks’ as intrusive and unnecessary, that people dislike changes to their home, and that many have a perception of low risk (Clemson, Cusick and Fozzard 1999; Simpson, Darwin and Marsh 2003).’ **^43(p.462)^**

‘Participants were asked to identify, from a list of eighteen events, which of the events they believed would occur in their lives within the next five years […] The number one risk indicated in the survey was injury from a fall, which was chosen by 91 percent of the seniors surveyed.’ **^51(p.18)^**

‘Perceptions of falling varied. On the one hand, falling was perceived as something inevitable and logical, while on the other hand, people had difficulties accepting it. Some had the impression that all older people fall. They had seen their mothers fall; therefore, falling was not something unusual. At the same time, they experienced that their bodies grew older, their reaction time became slower and their muscles strength weaker. These experiences strengthened their perception. One woman said: ‘‘When people get old, of course they fall. It is very natural. It has always been so.’’ For others, fall-accidents were embarrassing and shameful. Their feelings were reinforced if somebody saw them fall, and they changed their ways of living because they felt humiliated by falling.’ **^54(p.744)^**

‘Men were approximately 40% more likely to perceive that they had a low risk of falling compared with women. Younger respondents were more likely to nominate a low risk of falls—persons aged in their 60s were 70% more likely and persons aged in their 70s were 50% more likely than were persons in their 80s to perceive their risk of falling as low. Those with a partner and those with private health insurance were more likely to report a low risk of falls. Self-reported general health was strongly linked to perceived risk of falls—those who rated their health more positively were less likely to perceive a risk of falling. Those without a history of falls and fall-related injuries were less likely to be concerned about falling in the future.’ **^55(p.353)^**

‘Disagreeing or being unsure that “older people fall and there is nothing that can be done about it” was related to low self-perceived risk of falls. Those who identified prevention of falls as a low priority were also more likely to report that they had a low risk of falls.’ **^55(p.352)^**

‘Participants’ individual awareness of their own fall risks was an enabler to the belief that fall prevention behaviour was warranted (reflective motivation), “for myself, I have a bad case of kyphosis, sort of bent over when I walk, so I’m more likely to fall when I stand, but I make sure I keep both hands on my walker, that really helps me to feel balanced” (#106, intervention, male).’ **^64(p.9)^**

‘Researchers explored the influence of falls and fear of/concern about falling on participants’ awareness of falls and beliefs about their ability to prevent falls. Based on fall history, there were no significant differences in awareness or beliefs (Table 3). Participants who had fallen were more likely to have taken action to prevent a fall, although not all actions were significantly different based on fall history. More individuals who had fallen talked about falls to a family member or friend (ϰ2 [1, N = 267] = 6.1, p = 0.014) or their health care provider (ϰ2 [1, N = 267] = 5.8, p = 0.016), or made changes in their home (ϰ2 [1, N = 267] = 6.5, p = 0.010) than non-fallers.’ **^56(p.25)^**

‘Participants with fear of/concern about falling scores >6 (t [229] = –3.21, p = 0.002) were more likely to have talked to a health care provider about falling. Participants with higher concern were also more likely to have talked with family members or friends about reducing falls (t [207] = –4.372, p < 0.001). Participants who exercised had lower ratings of concern about falling (mean = 4.2, SD = 3.9) than participants who did not exercise (mean = 5.6, SD = 3.9) (t [211] = 2.22, p = 0.028). p.24-26.’ **^56(p.25)^**

‘Unless individuals are aware that adopting a particular behavior may reduce their risk of falling, or would not expect the adoption of the behavior to reduce perceived risk. Lack of awareness can result in a false sense of security whereby individuals who have not adopted a recommended behavior do not realize that failure to do so places them at greater risk.’ **^40(p.1316)^**

‘Findings suggest that awareness of recommended risk-prevention behaviors plays a critical role in risk perception.’ **^40(p.1316)^**

1. **Providers are primed to assess crime / fire safety on attending incidents (C). If providers learn to understand risk associated with health and wellbeing through training that promotes awareness and empathy, and where possible that links back to fire / crime safety, then they can better promote risk reduction behaviours (response) (M). Providers will approach risk in context of health (O).**

‘A primary concern of law enforcement officers is the constant precaution for life-threatening situations. They approach each situation mindful of immediate danger and their responsibility to enforce the law. After officers ensure safety, they evaluate the situation, such as an assault or felony, in terms of authority to arrest. A situation involving memory loss patients, however, should incorporate conciliatory procedures.’ **^50(p.360)^**

‘In recent years we have considered health in so much as it impacts on fire risk, however our access in Greater Manchester to more than 60,000 homes each year to carry out home visits affords us 60,000 opportunities to see, say and do more. We have therefore broadened the scope of our home visit so that these and other factors are considered in their own right i.e. from a health perspective as well as their impact on fire risk.’ **^65^**

‘It has provided training to professionals who go into people’s homes as part of their work, such as police, health professionals and council staff as well as fire officers, to enable them to spot dangers that might not traditionally be part of their work area and to explain to residents how they can get extra help and support.’ **^59(p.16)^**

‘Where the underlying causes of those fire risks relate to wider health and wellbeing issues, fire and rescue service staff can support people to remain independent in their own homes and reduce pressure on health and social care services. More than 80% of England’s fire and rescue services are developing similar approaches to tackling the underlying causes of fire risks.’ **^66(p.6)^**

‘The maturing approach to fire risk reduction that this pilot highlights supports the realisation among fire and rescue services that a single visit to a home solely to consider generic fire risk is also an opportunity to address the underlying causes of fire risk that are also wider health and being issues. Acceptance among recipients: little resistance to FRS staff undertaking the visit; fire/home safety clearly a selling point.’ **^66(p.6)^**

‘Older people are at an increased risk of both experiencing an accidental domestic fire [2] and falling [3]……The highest percentage of fatalities from fire occurring in the over 60 year age group [5], predominantly living in deprived areas……Factors increasing an older person’s risk of falling include: advanced age; reduced lower limb strength; balance deficits; history of falls; multiple and specific culprit medications, particularly sedatives; visual impairment; and cognitive impairment [3,6]. Similarly, reasons for the disproportionately high number of injuries and fatalities from fire in older age groups include physical and cognitive disabilities such as mobility problems; frailty; dementia; and medication use……..old housing [8] and single person households identified as risk factors. Three in five women aged 75 years or older live alone, [9] putting them at particular risk of harm from accidental domestic fires. Furthermore, a social class gradient to fire injury exists in the older population with people in lower income brackets at increased risk. [10] The consequences of fires and falls for older people are considerable and, as with the risk factors, are in many ways similar.’ **^61(p.2)^**

## Managing risks

1. **Older adults expect to discuss safety (C). Offering the opportunity to identify hazards and/or apply practical measures and/or model positive behaviours to maintain safety met their expectations and supported own sense of capability (M). Improved sense of control of own safety (O).**

‘Concern about safety in the home and a desire for independence were primary motivators for the older adult participants that Outreach Team members discovered.’ **^45(p.37)^**

'There was an increase in participants’ self-efficacy to prevent a fall and also to engage in falls prevention behaviors. Furthermore, participants’ sense of control over their preventive behaviors increased, or perceived barriers to engage in preventive behaviors decreased, after participating in the program.' **^45(p.42)^**

‘Perceived efficacy to prevent falls (p = 0.047), self-efficacy to engage in safety precautions to reduce the chances of a fall (p = 0.038) and perceived control over engaging in preventive behaviors increased (p = 0.000) between baseline and follow-up.’ **^46(p.1)^**

‘Perceived efficacy to prevent falls increased among the participants from an average of 2.56 at baseline to 2.75 (indicating an average between “somewhat” to “very confident”) at follow-up (p=0.05) (Table 8). Similarly, self-efficacy to engage in “safety precautions” to reduce the chances of falling increased from baseline to follow-up, with average responses of 2.97 and 3.12 at baseline and follow-up, respectively. Perceived control over engaging in preventive behaviors also increased from an average of 2.50 (between “to some extent” and “to a great extent”) to 3.18 (above “to a great extent”).’ **^45(p.27)^**

‘There is evidence that the pilot resulted in short-term outcomes for beneficiaries as a result of the home visit. This includes increased awareness by vulnerable people of some of the risks they may face during the winter period. MI data shows that over a third of households received IAG on the risks around falls and social isolation during the home visits and a quarter of beneficiaries recalled that the home visit had improved their awareness of these risks. Around one in ten beneficiaries reported improvements in awareness of the risk of falls, stating that the home visits identified and addressed hazards and informed them of how to reduce the risk of falling when on the move.’ **^66(p.36)^**

‘Most beneficiaries reported that having the fire service conduct a home visit gave them ‘peace of mind’ or reassurance that they were now in a safer home environment.’ **^66(p.37)^**

‘Motivation for participants to undertake fall prevention activities was influenced by their perceived loss of function and mobility. This participant who reported a serious falls injury, attributed the cause of the fall to external environmental factors, and continued to believe that fall prevention was beyond his control, “before going to hospital, I never thought about falls at all, because I could get around pretty well and it didn’t occur to me that a fall would happen” (#53, intervention, male).’ **^64(p.9)^**

‘“I put a ‘2’ for the safe from a fall questions because of my medical problems. I don’t think I can do anything to stop my knee collapsing and therefore falling….”‘ **^70(p.9)^**

1. **Older adults value independence (C). Offering a mixed set of accessible and convenient strategies such as information leaflet (cognitive), home modifications (physical), social support (socio-emotional), sign-posting (resource) promotes choice and sense of autonomy that enable recipients to determine cost-benefit of engagement against threat to independence (response) (M). Choice reduced sense of risk to independence (O).**

‘Specifically, emotional support, instrumental support (e.g., help with cleaning, shopping, making environmental modifications) and information support (e.g., information about how to reduce risks) are important in falls and fire prevention and maintaining autonomy in their homes.’ **^40(p.719)^**

‘A couple of Outreach Team members had to overcome hesitation from older adults about inviting them into their homes: "…they were a little bit skeptical at first about us coming into their homes and trying to tell them what to do but we just went there and, “Today we’re not here to judge you or we can’t force you. We’re just here to help you try to make things better. Keep you in your home as long as we can.” They realized that so they were good with it.’ **^45(p39)^**

‘We found testimonials from older adults who were already participating in the program to be valuable in recruiting additional participants, as well as recruiting via trusted community members, such as healthcare providers. It was important to emphasize the connection between the Remembering When™ program and maintaining independence, which is a key concern for many older adults, and to assure older adults that the aim of the program was not to judge or criticize.’ **^45(p.44)^**

‘Concern about safety in the home and a desire for independence were primary motivators for the older adult participants that Outreach Team members discovered.’ **^45(p.37)^**

‘The prospect of enhanced mobility in the context of fire risk may be more motivating, resonating with increasing independence.’ **^58(p.398)^**

‘During my introduction, which stressed that I was interested in the fire service’s prevention work, she said simply “I wouldn’t let them in the house”, going on to explain that this was because they would tell her to stop doing things.’ **^52(p.131)^**

‘Engaging with public services is not seen as being without risk. Some of those risks have already been discussed—that of being harshly judged, for example, or that of being targeted for having contact with authority. The perception of negative consequences of engagement goes wider than these issues, however. In particular it concerns the potential for contact with one agency to lead to less desirable contact with another agency. As such it is closely related to the topic of the fire service being seen as part of the wider state (Section 8.4).’ **^52(p.164)^**

‘A common theme amongst the people I spoke with was a sense of feeling judged, or a desire to avoid being judged. Judgement might come from many sources—from people outside of the area, from service providers, or from neighbours. …….. This sense of being judged was always associated with negative feelings, often anger, and I argue here that if the sense of being judged is associated with engagement with public services it is likely that people will seek to avoid engagement in order to avoid feeling judged.’ **^52(p.146)^**

‘Being told what to do (theme) p.152 [Barriers to engagement]: At a ‘Tea and Talk’ session early in the project Ruby talked specifically about the fire service and prevention work, telling me that she would not let fire crews in the house because they would tell her what she was doing wrong and that she needed to change things. At our first encounter Connie highlighted a similar point, telling me that to work in this community it is no good coming in and telling people what to do, rather there is a need to build empathy and trust.’ **^52(p.152)^**

‘Our findings suggest that older people do not reject falls prevention advice because of ignorance of their risk of falling, but because they see it as a potential threat to their identity and autonomy.’ **^73(p.508)^**

# INTERVENTION FLEXIBILITY

## Supporting individualisation

1. **Lack of awareness among providers about older adults’ existing knowledge / behaviours (C). In the absence of mutual dialogue between providers/recipients, providers cannot exploration of existing behaviours and enable the tailoring of advice and sign-posting (response) (M). Staff described "nitpicking" which may undermine the programme and be disempowering (O).**

‘Many older adults who were recruited to participate were already knowledgeable of fire and fall prevention practices: …most of them were really on top of things, I mean they really were. We nitpicked the residents as much as we could. We found two or three things that could be changed but like I said if you can change just one two things that really makes my day.’ **^45(p.37)^**

‘More than half of the participants used non-slip mats in the bathtub and on shower floors (67%), had grab bars on the walls next to the bathtub, shower and/or toilet (56%), and used rubber, non-skid pads on throw rugs (or had no throw rugs) (60%) prior to the RW program.’ **^46(p.721)^**

‘A common characteristic of interventions that may widen socio-economic inequalities in health appears to be ‘a reliance’ on self-directed behaviour change (White et al., 2009), as is the case with the Safe & Well visit. Inequalities may be introduced at different stages; such as in uptake and engagement, and in how individuals respond to an intervention. White et al.(2009) note that “the problem with ‘one-size-fits-all’ interventions has been recognised” and that interventions tailored to the needs of sub-groups within a target population may be more likely to result in outcomes that are more equitable.’ **^67(p.51)^**

1. **Providers trained to deliver a novel intervention (C). Providers trained to deliver an intervention as prescribed without adequate knowledge and experience to contextualise (resource) contributed to a lack of autonomy in applying own judgement (response) (M). Staff continued to conduct activities during home visits despite such activities seeming unnecessary or impractical, which caused frustration/unease among providers and recipients. (O)**

‘A few staff reported that they found that the test was sometimes unnecessary or impractical. For example, staff reported that they were often able to establish the vulnerability of a person by observing how quickly they answered the front door and watching them move around the house, as well as when they were developing an escape route in case of fire with the beneficiary. In addition, some staff found it impractical to roll out 3 metres of measuring tape, especially in properties which were very small or had limited space.’ **^66(p.33)^**

‘Some staff were concerned about how they could ensure that referrals had addressed people’s needs and felt that they should have feedback from the pilot management team about the actions taken by partner organisations once referrals had been made. Most frontline staff reported that they had little to do with the referral pathway, particularly firefighters. **^66(p.35)^**

‘The majority of participants stated they felt they had insufficient training to deliver Safe and Well Checks to the high quality they wanted. The amount of training they reported having had ranged from none (one participant) through a few hours to a few days. Frequently this training was after they began delivering Safe and Well Checks: ‘I’m not saying the Safe and Wells isn’t a good thing, but I really think before it was bought in it was…we should have a lot more training than we did have, I really do’—ID14. The firefighters said that the training often focused on how to complete the form documenting their conversation with service users, rather than the complex communication required to carry out a Safe and Well Check and that ‘If we get the right information to give I would feel happier doing it and I think it would be a good thing’—ID5.’ **^44(p.596)^**

## Harnessing support networks

1. **Older adults living at home without prior engagement with support services (C). By assessing individual needs and providing opportunities to connect with support services (resource), awareness and access to support can be improved (response) (M). Acceptance of information / sign-posting is widely accepted but a high rate of decliners to service engagement / referrals is reported (O).**

‘No changes were observed for discussing falls prevention with health care providers or senior center staff.’ **^45(p.33)^**

‘71.1% refused the opportunity for either NHS support or Strength and Balance Classes. Of those who accepted intervention, 26.9% were attending Strength and Balance classes and 2.0% were referred to the NHS Community Care Team.’ **^67(p.41)^**

‘Information was only available about onwards referral so information is lacking about service uptake; 24.0% were referred to the falls team and 28.0% were referred to Strength and Balance classes; 48.0% of referrals were subject to ‘other’ actions.’ **^67(p.41)^**

‘Following telephone triage, 50.6% of referrals did not meet the referral criteria for the service. 49.4% received a multifactorial falls risk assessment in the home; 50.0% of whom were discharged and 28.9% of whom received support (14.3% of all the referrals received).’ **^67(p.41)^**

‘“…We are looking at the onward acceptance of referrals as we know there is a big drop off between referral and acceptance into services particularly for falls. Therefore we are not sure the pathway is good enough at the moment and we may be missing opportunities”. (Local authority, Merseyside)’ **^67(p.37)^**

‘Around two-thirds of people interviewed reported receiving information for them to contact another agency or were informed that they should expect to hear from another organisation to address a particular issue. They reported that they had either been followed up with the other agency or were contacted by another organisation. All of those who received a referral reported that this was appropriate and done with their consent. A small number of people reported refusing a suggested referral. This was largely because they did not feel it was necessary or because they believed they were already accessing the necessary support to manage their needs. The pilot also facilitated beneficiaries in receiving support from other services. MI data shows that 3,376 referrals were made to partner organisations as a result of the pilot in order to provide support to beneficiaries. Over a third of beneficiaries reported that they recalled being referred or provided with contact details for further support at the time of the home visit from partner organisations, including Age UK, local authorities and falls assessment teams. There is some evidence that the support received from other services resulted in outcomes for beneficiaries in the medium term. Nearly a fifth of beneficiaries reported (at the time of the interview) that they had been contacted and assessed by partner organisations as a result of the home visit to address their support needs, including needs around falls (for example, falls assessment teams, physiotherapist) and social isolation (for example, local befriending service). One in ten beneficiaries reported improvements to the home environment to support their mobility needs and reduce the risk of falls, such as the installation of hand rails and other home adaptations. As one beneficiary described: “I have exercises, a seat in the shower, and also I have got a toilet frame so can I can lean when I get up which has helped a lot…as a result of visit from the physio…We couldn’t believe it was all the fire service that [initiated] it”. A few beneficiaries also reported that they had received support to reduce their social isolation and were now receiving visits or talking to people over the phone on a regular basis. Around three-quarters of beneficiaries stated that they received their support with flu vaccinations from health services and received their vaccination prior to the home visit taking place.’ **^66(p.37)^**

‘Fire perspective: John Beard said “We are making the most of our time when we are invited into people’s homes. If we can take the opportunity to pass on messages and advice from partners we will do so. We go further though as we are actively signposting those in need to the services most able to provide the early intervention”.’ **^60(p.12)^**

‘“Vulnerable residents who were being rescued by the fire service were often known to other services, such as social services, health and GPs. What had been missing was the opportunity to share information about who each service recognised as in need of help, she said.’ **^60(p.11)^**

‘One elderly resident who was helped by SAIL said it had “unleashed an army of people” to see her.‘ **^60(p.10)^**

‘"Along with the usual safety checks carried out by GMFRS’ firefighters and community safety teams we added an assessment on the risk of falls for people aged over 65 and referred those at higher risk to the health service’s falls prevention team.” Another woman who was referred to the falls team after having a home safety check was 85-year-old Margaret Conroy, also from Bury. Mrs Conroy says: “After having smoke alarms fitted by firefighters during a home safety check a therapist from the health service came to see me and talked over an exercise plan. I now go to the gym several times a week to strengthen my legs as I have suffered break injuries over the past few years.” “I use two sticks to help me get around and am hoping to get down to one stick when my legs are stronger.”‘ **^59(p.16)^**

1. **Engagement with public service personnel (C). Engagement provides an opportunity for social interaction with a trusted individual, which members of the public could gain reassurance and social support from (response) (M). Enhances sense of social support (O).**

‘“The interview was conducted very professionally. Comforting to know someone cares, especially for elderly people living alone and of course fire alarms for free” (Postal survey respondent).’ **^67(p.22)^**

‘Nine [out of 55] participants specifically mentioned that they appreciated interacting with and learning new information from the Outreach Team members who conducted the group presentations and home visits. As one participant stated, “I really enjoyed the visit from the fireman… I thought it was very useful and he’s a lot of fun, too.”’ **^45(p.33)^** *Ellipses added*.

'Participants reported increased informational support from the people around them about falls and fire prevention, likely due to becoming acquainted with the local fire service personnel through the program and who often indicated they would continue to be available to the participants. This finding may point to one of the unintended positive consequences of the RW program that, in addition to increasing preventive behaviors among older adults, RW may enhance overall social support network systems among community dwelling older adults.' **^46(p.725)^**

The number of participants who reported discussing falls and fire prevention with their family and friends increased from baseline to follow-up in both study arms. At baseline, 34% had discussed falls prevention with family and friends, whereas 60% reported discussing this at follow-up (Table 10). **^45(p.33)^**

‘Engagement in discussions about falls and fire prevention, however, was not accompanied by an increase in perceived social support related to falls or fire prevention in our study. What we observed were increases in general perceived social support, such as emotional and instrumental support.’ **^46(p.725)^** [UNINTENDED MECHANISM AND OUTCOME RELATED TO SOCIAL SUPPORT NETWORK]

‘Social networks with friends did not show evidence of reducing falls, however, it did keep older adults socially active which can enhance health behaviors relevant to falls prevention [41]. It may be that RW can enhance overall social support systems of older adults that likely can contribute to their overall well-being [42, 43].’**^46(p.725)^** [UNITENDED MECHANISM AND OUTCOME RELATED TO SOCIAL SUPPORT]

‘A similar increase was also reported for general social support perceptions, such as emotional support, instrumental support, and support when sick or injured. Participants reported discussing falls or fire prevention with others in their lives, including family and friends, where at baseline, 34% had discussed falls prevention with family and friends and 60% reported discussing this at follow-up.’ **^45(p.30)^**

'Most (n=47) of those participants explicitly expressed general satisfaction with their participation in the study and Remembering When™, saying, for instance, “I’ve enjoyed this” and “it was fun.”’ **^45(p.33)^**

‘No significant changes were seen for perceived social support participants received from others in reducing a fall’ **^45(p.30)^**

‘MI data shows that over a third of households received IAG on the risks around falls and social isolation during the home visits and a quarter of beneficiaries recalled that the home visit had improved their awareness of these risks.’ **^66(p.36)^**

# SERVICE INTEGRATION

## Within service culture, capacity and context

1. **Providers operate within an established team culture (C). Providing intervention training to “non-traditional” staff that incorporates opportunities to interact with other learners, to ask questions and reinforce collective effort / teamwork, supported preparation, provided reassurance and confidence (M). Staff that accessed online learning prior to intervention delivery reported poorer engagement, lower confidence and lower sense of preparedness than those who received face-to-face training (O).**

‘Frontline staff that had face-to-face training were generally more positive than staff who had attended webinars.’ **^66(p.25)^**

‘In Greater Manchester, CSAs received face-to-face training, whilst the fire crews received training via the webinar. Fire crew staff that were interviewed generally found the webinar not very useful and would have liked to have had the opportunity to access face-to-face. Greater Manchester operational leads and key delivery staff reported that, following feedback from crews, they introduced a series of question and answer sessions, specific email and online access to advice and guidance, and a direct line to support frontline staff and address concerns. A total of 1,200 staff received training to deliver the intervention. Staff reported improved skills and knowledge in relation to falls prevention, cold homes, flu vaccinations and social isolation. Face-to-face training was more effective than webinars at achieving this. Around two-thirds of survey respondents from Greater Manchester indicated that the training had not sufficiently prepared them to conduct all aspects of the home visits, including assessing risks, providing IAG, addressing issues within the home, and making referrals, in relation to falls, cold homes, flu and social isolation.’ **^66(p.25)^**

‘Most of the frontline staff interviewed from Staffordshire and Gloucestershire said that on the whole, they felt the training they had received prepared them for delivering the home visits and knew where to go should they require any additional information. A few staff in Gloucestershire believed that they would have benefited from further training sessions to refresh everything they were meant to cover during the visit.’ **^66(p.24)^**

‘“When accompanying fellow advocates on visits (due to lack of vehicles) and seeing how they deliver safe & well, my confidence has improved. Also over time getting used to the new format. My confidence has improved from regular CPD days too.” (Advocate, more than 100 Safe & Well visits made)’ **^67(p.18)^**

‘Teamwork has a long-standing culture in the fire service and moving to a solo responder model was considered a real obstacle. Because single responders are counter to active team maintenance, several fire and rescue services using this model provided another firefighter. In some cases the fire service paid for the second firefighter to attend which was seen as unsustainable in the long-term.’ **^72(p.56)^**

‘Effective training in a closed society such as law enforcement requires that groups who are respected by police officers reinforce the training program.’ **^57(p.270)^**

‘A letter of support from the attorney general: Recommended in-service training by local Alzheimer chapter representatives or police-training officers.’ **^57(p.271 – Table 1)^**

‘Role play scenarios elicited officers’ knowledge and demonstrated their ability to identify persons with AD and to demonstrate the steps involved in contacting the Safe Return program.’ **^57(p.272)^**

‘A survey of law enforcement personnel found that police training officers are viewed by other officers as role models.’ **^62(p.319)^**

‘To help law enforcement officers empathize with the memory loss patient, a discussion exercise is implemented. Participants identify and give priorities to significant factors in their lives and discuss their feelings of loss (Kubler-Ross, 1969, 1975). Patients’ behavioral problems, such as confusion and inability to communicate, and stressors in caregivers are discussed. Family issues, such as strained relationships, legal concerns, and the need for information are explored. Role playing highlights the problem of traditional interventions, such as high visibility of the patrol car and lights frightening the memory loss patient. Officers are encouraged to try different conciliatory approaches, such as calm, simple, repeated phrases, and referrals to appropriate community resources.’ **^50(p.359)^**

1. **Capacity and resource available within service (C). Where services have dedicated roles and experience to deliver “non-traditional” intervention, delivery of intervention can be delivered to high standards (M). Services more likely to achieve targets and not over-burden staff.**

‘One of the major barriers Outreach Teams experienced in completing the study activities, which included Remembering When™ program delivery and completing data collection instruments, was limited time and staff support. One Outreach Team member described the issue as follows: "I mean I really enjoyed it, but it was we were trying to get a lot of visits in a short amount of time. For me, being really the only person doing them, I felt a little scatterbrained [laughter] or a little hectic some days trying to make sure I was getting everything done."‘ **^45(p.40)^**

‘Some “teams” operated with only one active member who conducted all home visits and group presentations, whereas others had two people engaged. Even for some two-member teams, scheduling home visits and delivering them was difficult to do alongside other fire department duties. Lack of time and a staffing shortage contributed to one Outreach Team being unable to complete the study. A representative of that team stated that the study time commitment was not clear at the beginning and wished that the research team had given them a better idea of how much time would be required.’ **^45(p.40)^**

‘Gloucestershire managed to achieve around two-thirds of their target home visits (68%) over the course of the pilot. Operational leads and delivery staff reported that challenges in getting the database up and running (in order to capture the information required for the pilot), and limited capacity to train large numbers of staff at one time in order to deliver the home visits, contributed to delaying the full implementation of the pilot by a month. This is illustrated in Figure 2, which shows fewer home visits in the first month compared to other months.’ **^46(p.29)^**

‘Existing experience and capacity to deliver home visits, combined with an established referral pathway, enabled pilot areas to hit the ground running. Staffordshire had already been delivering a similar pilot to the Winter Pressures Pilot (under the SAfER pilot) and were able to draw on this experience and local networks. It is likely that this helped the FRS to exceed its target number of home visits and reduced the chances of setbacks and delays. In addition, having sufficient time to plan and prepare for delivery was important to the smooth implementation of the pilot**.’ ^66(p.45)^**

‘Each partner in this initiative had clear roles and responsibilities. The training officer in each local police department promoted and set up the training, completed the evaluation instruments, and saw that resource materials were appropriately distributed to police officers and dispatchers. The health educator from NNJCAA worked with the chapter to develop and deliver an expanded training program and resource materials, designed process and impact assessment instruments, and provided positive recognition through on letters of commendation and certificates of achievement.’ **^57(p.272)^**

1. **Implementing a new intervention in a local context (C). Incremental roll-out of interventions allows for any problems to be overcome and appropriate improvements and alterations to be made to the approach without too much disruption to the service. Small scale implementation was more effective (M). Large scale pilots underachieved on target numbers for home visits (O).**

‘There is an indication that implementation is more effective on a smaller scale, across a single area, compared to large scale implementation across a metropolitan area, with a diverse population. Both Staffordshire and Gloucestershire delivered the pilot incrementally over a smaller geographical area than Greater Manchester and reported fewer challenges and problems in delivering the home visits. The benefits of this approach were that they could learn from problems as they went along and alter parts of the pilot without too much disruption.’ **^66(p.45)^**

‘Little opportunity to give their feedback to managers in the service about the expansion of their roles: ‘Feedback is never really something we had the opportunity to give’—ID16 and that it might not have an impact even if it is given: ‘if I’m honest I think its futile’—ID11. Many participants reported a suspicion that the quality of the Safe and Well Checks was not monitored: ‘it just seems to be about numbers at the minute…say if somebody at the top wants to say ‘we did this many Safe and Wells,’ and they’re not really about Safe and Wells’—ID20 and that there wasn’t enough regard for the practicalities of making the changes to their roles. “We were never told why it had to be done straightaway. The same with Red One stuff, it was never said why we had to start it bang on the date we did. Why not get things right and then we’ll ease it in?” ID18. There was a suspicion that the fire service was being called upon to support public services that were not being resourced appropriately, e.g. stating that they were ‘making up for all the other services that are underfunded’ ID9.’ **^44(p.596-7)^**

## System-level collaboration

1. **Operating within an established/dynamic health and social care network (C). The partnership between several organisations (resource) should enhance the success of any programme for older adults HOWEVER having shared interests is not sufficient in and of itself. Partners need to identify aligned objectives, show leadership, share information and expertise and engage in communication at all levels. Frustration will arise if partnerships are not successful (M). Variability in collaborative working (O).**

‘Nearly all partner organisations indicated that the pilot had led to improved communication and relationships between themselves and the FRS and they intended to further develop joint working in the future.’ **^66(p.9)^**

‘Partners generally supportive of winter pressures components and that targeting complemented their own work. They felt that greater collaboration in the development of the pilot could have: enabled the FRS to draw on greater local knowledge about the needs of vulnerable people within local populations with the potential to develop and improve data and information sharing processes between the FRS and partners.’ **^66(p.23)^**

‘Engaging with partners from the planning stages of the pilot was also important. The majority of partners and FRS reported that they felt the pilot would have benefited from greater collaboration at the beginning, potentially through a pilot area steering group, to better establish data sharing mechanisms between the FRS and partners to support the referral pathways, but also support the FRS in reaching the right people.’ **^66(p.10)^**

‘A multi-partner steering group should oversee the establishment of the Safe and Well visit within local areas. This should be carried out as part of a wider system approach to address health improvement and reduce demand on public sector services operating in the home setting. It will also improve the alignment of the FRS with other services, and vice versa.’ **^66(p.12)^**

‘Using data about local populations to identify and target vulnerable households takes considerable time and resource. Involving local partners and agencies, with their specialist knowledge of vulnerable people within local areas, would be beneficial.’ **^66(p.45)^**

‘All patients aged 60 years and older attending the four Falls clinics receive from their clinic nurse the ‘Fire Safety in the Home’ booklet [30] which contains information on fire risk reduction and the Home Fire Safety Visit scheme. Clinic staff have been trained to use a standardised message about the booklet and importance of the Visit, to give participants an information leaflet about the intervention, and to mark on the daily clinic list all patients who have received this information. Patients are also given a Home Fire Safety Visit freepost card, to which they (or their carer) are asked to respond by booking a Visit by the Fire Brigade. The service is free to access and receive. An additional Freepost card is appended to the clinic letter posted to every new patient attendee after their appointment.’ **^61(p.5)^**

1. **Acknowledging organisational knowledge (C). Having the opportunity and agreement to share local organisational knowledge and awareness between partners, reaffirms collaborative trust and respect (M). Likely to enhance reciprocity (O).**

‘Humberside FRS have played a leading role in Hull 2020, with a Group Manager leading the frail and elderly work stream and they have seconded a Station Manager full time into the CCG. This secondment is designed to improve the already strong links with the CCG, and enable HFRS and the CCG to better understand each other’s work. The SM has led on a number of the collaboration projects mentioned above, and acts as a conduit and “translator” between the worlds of health and fire. His brief is to also explore what else could be provided with our other CCGs, not just Hull, and this is already starting to deliver useful outcomes.’ **^48(p.2)^**

‘Although this would not always be feasible, the Chief Executive of Hull CCG also spent a full night shift on a fire station and rode with the crew, which improved her knowledge of what the FRS did and could do, and gave her a good appreciation of the challenges FRS face.’ **^48(p.2)^**

'The most effective method to reach the target population was to combine resources with a coalition of organisations who were providing services to older adults' **^49(p.3)^**

‘For example, Age UK is supporting by sharing knowledge, resources and expertise with the PHE/CFOA advisory board and fostering support amongst local Age UKs who can offer FRSs useful assets and expertise to achieve success. Support knowledge exchange and reciprocity.’ **^65^**
